# Maize yield estimation in Northeast China’s black soil region using a deep learning model with attention mechanism and remote sensing

**DOI:** 10.1038/s41598-025-97563-6

**Published:** 2025-04-15

**Authors:** Xingke Li, Yunfeng Lyu, Bingxue Zhu, Lushi Liu, Kaishan Song

**Affiliations:** 1 School of Geographic Science, Changchun Normal University, Changchun, 130102 China; 2 State Key Laboratory of Black Soils Conservation and Utilization, Northeast Institute of Geography and Agroecology, Chinese Academy of Sciences, Changchun, 130102 China

**Keywords:** Yield prediction, One-dimensional convolutional neural network, Bidirectional gated recurrent unit, Attention mechanism, Agroecology, Climate sciences, Ecology

## Abstract

Accurate prediction of maize yields is crucial for effective crop management. In this paper, we propose a novel deep learning framework (CNNAtBiGRU) for estimating maize yield, which is applied to typical black soil areas in Northeast China. This framework integrates a one-dimensional convolutional neural network (1D-CNN), bidirectional gated recurrent units (BiGRU), and an attention mechanism to effectively characterize and weight key segments of input data. In the predictions for the most recent year, the model demonstrated high accuracy (R² = 0.896, RMSE = 908.33 kg/ha) and exhibited strong robustness in both earlier years and during extreme climatic events. Unlike traditional yield estimation methods that primarily rely on remote sensing vegetation indices, phenological data, meteorological data, and soil characteristics, this study innovatively incorporates anthropogenic factors, such as Degree of Cultivation Mechanization (DCM), reflecting the rapid advancement of agricultural modernization. The relative importance analysis of input variables revealed that Enhanced Vegetation Index (EVI), Sun-Induced Chlorophyll Fluorescence (SIF), and DCM were the most influential factors in yield prediction. Furthermore, our framework enables maize yield prediction 1–2 months in advance by leveraging historical patterns of environmental and agricultural variables, providing valuable lead time for decision-making. This predictive capability does not rely on forecasting future weather conditions but rather captures yield-relevant signals embedded in early-season data.

## Introduction

Agricultural production is vital to human livelihoods and well-being^1^. Crop yield forecasting can help farmers adjust field management measures in time and assist the government in formulating food security policies, adjusting regional planting structure and developing crop production and yield monitoring systems^2^.

Process-based crop modeling and empirical regression are the two main approaches currently used to predict crop production using satellite imagery^3,4^. By connecting data from satellites, process-based crop models can simulate continuous crop development, growth, and yield using algorithms and particular parameters relevant to crop growth^5^. By using remote sensing data assimilation to the regional scale crop growth model, to predict crop yield^6^. In recent years, the most common crop models combined with remote sensing data are WOFOST^7^, DSSAT^8^and AquaCrop^9^. Most process-based crop models require data on environmental variables throughout the phenological period to complete the simulation, which makes yield assessment difficult to apply at regional scales. By contrast, the empirical regression methods have relatively liberal requirements for input variable data. They are usually based on a given time or stage of vegetation index or the experience of the relationship between environmental factors and the actual measurement output to predict crop yield. Since it is not limited by specific crop parameters and determined growth time, it is widely used for yield prediction of various crops^10,11^. Compared with crop models that require whole-period information, this method is more direct for predicting early crop yield^12^.

Machine learning (ML) can effectively overcome the shortcomings of traditional empirical methods using satellite images to predict large-scale crop yield. It conducts analysis and learning on the data, and then utilizes the learning outcomes to make decisions in accordance with the input variables^13,14^. Machine learning algorithms, including multi-layer perceptron (MLP), support vector machines (SVMs), and random forests (RFs), have been extensively utilized in the domain of agricultural yield forecasting^15–17^. This is attributed to their capacity to capture the complex, nonlinear relationships between environmental variables and yield outcomes. Nevertheless, the efficacy of these machine learning models is contingent upon the quality and volume of data available. The manual selection and design of feature representations are also critical. In scenarios where data is scarce, these models are susceptible to overfitting or underfitting, which can impede their effectiveness in addressing nonlinear challenges and in accurately forecasting crop yields across extensive geographical areas^18,19^.

Deep learning (DL) models excel in the extraction of intricate and precise features through end-to-end training across multiple hidden layers. Empirical studies have indicated that DL approaches often surpass conventional machine learning techniques in terms of accuracy in crop yield prediction^12,20–22^. Among the myriad of deep learning models, Convolutional Neural Networks (CNNs) and Recurrent Neural Networks (RNNs) have emerged as particularly prominent architectures^23^. CNN achieves good yield estimation performance in remote sensing image information extraction and crop yield estimation by virtue of its powerful feature extraction ability^24^. RNNs are particularly adept at processing time series data due to their ability to capture temporal dependencies. Within the RNN framework, Long Short-Term Memory (LSTM) networks and Gated Recurrent Units (GRUs) have been developed as advanced variants^25^, solving the problem of gradient disappearance and gradient explosion. Cho^26^introduced the GRU, which offers a more streamlined architecture than LSTM while retaining its capability to capture and maintain long-term dependencies. Schuster and Paliwal combined the output of forward processing sequence and reverse processing sequence, and improved the model to BiGRU by using the contextual information of sequence data^27^. The results show that BiGRU performs better than standard GRU and traditional machine learning algorithms in time series data prediction^28^. However, it should not be ignored that in BiGRU, the model mainly relies on fixed-size context vectors when processing sequences, which makes the model may lose some important information in long sequences. In contrast, attention mechanisms have demonstrated the ability to more effectively capture global information. By dynamically assigning varying weights to different positions within a sequence, these mechanisms enable the model to focus on the most salient aspects of the data, thereby enhancing the model’s overall performance^29^. Based on the excellent performance of CNN, GRU and attention mechanism in previous predictions, the deep learning framework (CNNAtBiGRU) constructed by combining CNN with attention mechanism and bidirectional GRU has been used in energy consumption^30^and shale oil production^31^. However, its application to crop yield prediction has not been systematically investigated, suggesting that the framework’s potential in this area remains largely untapped^32^.

In crop yield prediction, existing deep learning frameworks such as CNN or LSTM face limitations in effectively combining spatial and temporal features and fully utilizing diverse input variables. To address these challenges, we introduce CNNAtBiGRU, an architecture that integrates a one-dimensional CNN, BiGRU, and an attention mechanism, optimizing spatiotemporal data modeling while enhancing prediction accuracy and interpretability. By combining these techniques, CNN captures spatial characteristics from satellite imagery, BiGRU extracts bidirectional temporal dependencies in time-series data, and the attention mechanism selectively emphasizes critical features, ensuring more reliable yield estimation. This study builds upon this framework to achieve the following objectives: (1) Propose a novel deep learning model (CNNAtBiGRU) to improve the accuracy of real-time maize yield predictions by optimizing the integration of spatiotemporal data from remote sensing, climatic, and soil variables.(2) Compare the CNNAtBiGRU framework with five other yield estimation methods, spanning deep learning, machine learning, and linear regression, to validate accuracy in forward and backward time-series predictions.(3) Assess the contributions of diverse input variables, identifying the most effective combinations for robust yield forecasting.(4) Examine the model’s adaptability in extreme climate scenarios, evaluating its performance in predicting maize yields under varying conditions and exploring trends and strategies for sustainable maize production.

## Materials and methods

### Study area

**Fig. 1:**
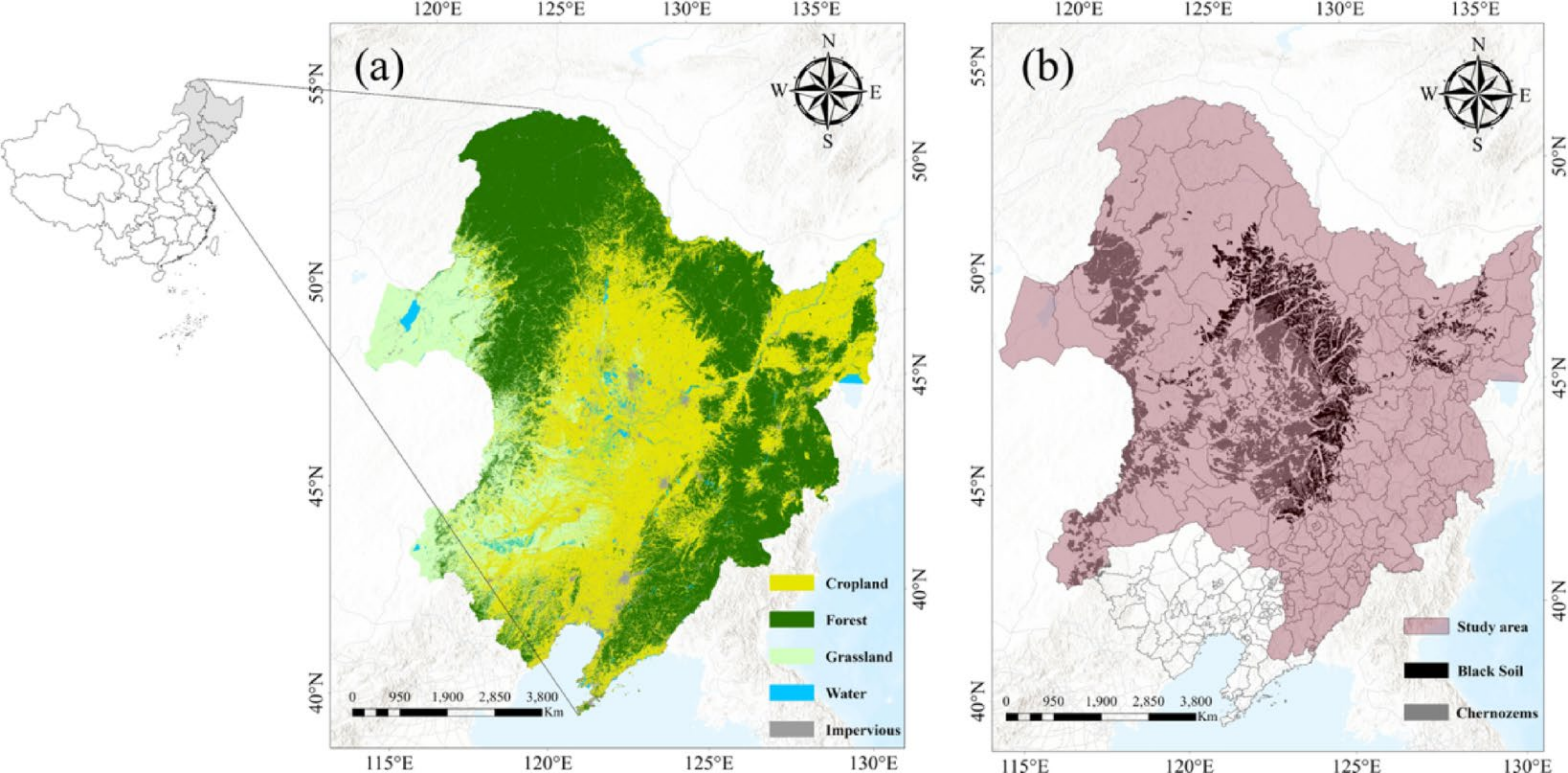
(**a**) Distribution of 30 m-resolution land cover type in 2022, (**b**) distribution of phaeozem and chernozem in Northeast China. (Base map data adapted from GS(2020)4619, http://bzdt.ch.mnr.gov.cn/. Map generated using ArcGIS Pro 3.1.6, http://www.esri.com).

The study area, situated in Northeast China (42°56’N- 50°03’N, 122°05’E- 128°12’E) (Fig. 1), covers approximately 1,030,000 square kilometers. Characterized by a temperate continental monsoon climate, the region has an average annual temperature of around 0.4 °C and receives an average annual precipitation of approximately 534 mm. The topography varies, with altitudes ranging from 250 to 450 m. Most of the farmland in the study area is used for planting maize, rice and soybeans^33^. The region’s soils primarily include phaeozem and chernozem, which are considered among the best options for maize cultivation due to their high fertility and rich organic matter content. In this study, from 258 county-level administrative regions, 201 typical counties with typical black soil areas that have continuously produced maize for more than 20 years during the 21 years from 2001 to 2021 were selected^34^. Maize cultivation in the region is mainly rain-fed, and irrigation is uncommon. Therefore, the crop model used in this study does not include irrigation scenarios.

### Data and pre-processing of input variables

We summarized the sources of maize yield at the county level, planting area, remote sensing variables, environmental variables, agricultural management data, agricultural modernization characteristics and geospatial data (Table 1). The data collection mainly uses the Google Earth Engine^35^. In this study, the nearest-neighbor interpolation method was used to resample the raster input variables to a uniform spatial resolution of 500 m using the Asia North Albers Equal Area Conic projection, and to crop them to each county administrative region. This procedure was applied to harmonize the different spatial resolutions of the data. The dataset encompasses a period from 2001 to 2021, comprising a total of 4,220 county-level administrative divisions.

**Table 1:** Input variables for maize yield prediction using the CNNAtBiGRU, CNNGRU, GRU, LightGBM, and LASSO models.

Category	Variables	Spatial resolution	Temporal Resolution	Source
Maize yield and planting area	County yield	County	Yearly	Agricultural Statistical Yearbook
	Planting area	30 m	Yearly	Peng, et al.^36^
	Maize yield in field	–	–	Field-surveyed data
Remote sensing variable	NDVI, EVI, GCVI, GNDVI, WDRVI, OSAVI	500 m	8-day	MOD09 A1 product
	SIF	0.05°	4-day	Tao, et al.^37^
	LST	1 km	Daily	MOD11 A1 product
Environmental data-climate	Climate data (Tmax, Tmin, Pre, Pdsi, Pet, Vap, Vpd)	4 km	Monthly	TerraClimate datasets^38^
Environmental data-soil	Soil properties (Texture, Clay, Silt, Sand, SOC)	500 m	5-year	Soil particle-size distribution dataset^39^
Agricultural management data	Consumption of chemical fertilizer	County	Yearly	Agricultural Statistical Yearbook; Government statistical bulletin
	Comprehensive mechanization rate	County	Yearly	
	Gross domestic product (GDP) grid	1 km	Yearly	Chen, et al.^40^
Agricultural modernization data (AgriMD)	Degree of fertilizer consumption (DFC)	500 m	Yearly	Silvestri, et al.^41^
	Degree of comprehensive mechanization (DCM)	500 m	Yearly	Yang, et al.^42^
Geospatial	Longitude, Latitude (Lon, Lat)	County	Yearly	

#### Maize yield and acreage

County-level maize yield data from 2001 to 2021 were obtained from the county-level agricultural yearbook(http://www.stats.gov.cn), measured in tons per hectare. An initial quality assessment was performed to detect and exclude outliers. Annual maize planting area data at a 30 m resolution for the same period were integrated with a previously published dataset by researchers^36^. County selection was based on the availability of complete production and acreage statistics for maize over 20 of the 21 years within the study period, at the county-level administrative district scale. Ultimately, 201 counties were included in the study each year, representing approximately 80% of the maize planting area in the typical black soil regions.

To integrate the high-resolution maize planting area data with MODIS-based remote sensing and climate datasets, a multi-step process was employed. First, the raw 30-m maize planting area data were aggregated to a 500-m grid by calculating the number of maize pixels within each cell—up to a theoretical maximum of 277 pixels per cell, depending on pixel alignment and coverage. Next, the proportion of maize coverage in each 500-m grid cell was determined by dividing the maize pixel count by this maximum value.In order to include only those cells that truly represent dominant maize-growing areas, a threshold of 80% was applied. This means that a grid cell was classified as a maize field only if the aggregated maize pixels accounted for at least 80% of the maximum possible. The choice of a 80% threshold was not arbitrary: it was derived from a visual interpretation of the distribution of maize pixel counts within grid cells^43^. This evaluation ensured that the threshold struck an optimal balance between retaining sufficient sample areas and minimizing the inclusion of mixed land-cover noise.While this resampling approach does introduce some spatial generalization and potential loss of fine-scale heterogeneity, it establishes a consistent and practical framework for integrating multiple data sources at a 500-m resolution.

#### Satellite products and environmental data

Seven satellite-derived vegetation indices (NDVI, EVI, GCVI, GNDVI, WDRVI, OSAVI, and SIF) and land surface temperature (LST) were selected to approximate aboveground crop dynamics, photosynthetic activity, and thermal conditions. These indices are detailed in Table 2, including their calculation methods and reference sources. The time-series vegetation indices were sourced from the 8-day composite surface reflectance product of the Medium Resolution Imaging Spectrometer (MODIS) version 6 (Terra/MOD09 A1) with a spatial resolution of 500 m. Although the MOD09Q1 product offers a finer 250 m resolution, we opted for MOD09 A1. This decision was based on its enhanced atmospheric correction, improved long-term temporal consistency, and more consistent provision of the necessary spectral bands, especially the blue and green bands that are critical for indices such as EVI, GCVI, and GNDVI in computing our full suite of vegetation indices^44^. Meanwhile, LST data were obtained from the MODIS Land Surface Temperature product (MOD11 A2) with a spatial resolution of 1 km and a daily temporal resolution. Sun-Induced Chlorophyll Fluorescence (SIF)^45^, a novel signal of photosynthetic activity that has gained prominence in recent years for crop monitoring and yield prediction, was extracted from the Global Spatial Continuous SIF dataset with a 4-day temporal resolution and a 0.05° global grid^46^. The full time series of these vegetation indices underwent harmonic regression analysis to derive monthly mean values for the growing season.

To capture diverse environmental conditions, additional data were incorporated, including minimum temperature (Tmin), maximum temperature (Tmax), precipitation (Pre), Palmer Drought Severity Index (Pdsi), potential evapotranspiration (Pet), vapor pressure deficit (Vpd), and vapor pressure (Vap). These environmental variables were sourced from the TerraClimate dataset, which has a spatial resolution of 4 km and a monthly temporal resolution^38^. All vegetation indices, LST, and environmental variables were resampled to a 500 m spatial resolution using the nearest-neighbor interpolation method to align them with the maize planting area raster data for consistent spatial matching.

As shown in Table 3, all the above-mentioned data, categorized as dynamic variables, were updated monthly during the maize growing season to capture temporal variations in growth conditions.

 –

**Table 2:** Vegetation indices and their calculation equations.

VIs	Abbreviation	Equation	Reference
Normalized Difference Vegetation Index	NDVI	$\:\frac{\text{N}\text{I}\text{R}-\text{R}}{\text{N}\text{I}\text{R}+\text{R}}$	Rouse, et al.^47^
Enhanced Vegetation Index	EVI	$\:\frac{2.5\times\:\left(\text{N}\text{I}\text{R}-\text{R}\right)}{\left(\text{N}\text{I}\text{R}+6\times\:\text{R}+7.5\times\:\text{B}+1\right)}$	Huete, et al.^48^
Wide Dynamic Range Vegetation Index	WDRVI	$\:\frac{0.2\times\:\text{N}\text{I}\text{R}-\text{R}}{0.2\times\:\text{N}\text{I}\text{R}+\text{R}}$	Gitelson^49^
Green Chlorophyll Vegetation Index	GCVI	$\:\left(\frac{\text{N}\text{I}\text{R}}{\text{G}}\right)-1$	Gitelson, et al.^50^
Green Normalized Difference Vegetation Index	GNDVI	$\:\frac{\text{N}\text{I}\text{R}-\text{G}}{\text{N}\text{I}\text{R}+\text{G}}$	Gitelson, et al.^51^
Optimized Soil-Adjusted Vegetation Index	OSAVI	$\:\frac{\text{N}\text{I}\text{R}-\text{R}}{\text{N}\text{I}\text{R}+\text{R}+0.16}$	Rondeaux, et al.^52^
Solar-Induced Fluorescence	SIF	-	Zhang, et al.^46^

$\:NIR,\:R,\:B$ and $\:G$ represent reflectance at near-infrared red, blue and green wavelengths, respectively.

**Table 3:** Summary of dynamic variables and processing.

Category	Features	Phenology	Composite method
Satellite data	NDVI, EVI, GCVI, GNDVI, WDRVI, OSAVI, SIF, LST	5 stages	Mean
Environmental data	Tmin, Tmax, Pre, Pdsi	5 stages	Sum
	Pet, Vap, Vpd	5 stages	Mean

5 stages represent the five key months of the maize growing season in Northeast China, specifically May, June, July, August, and September.

Understanding the foundational conditions that influence crop growth and yield is pivotal, And soil as a type of environmental data, plays a critical role in this regard. This study incorporated several key soil variables, such as soil texture, which denotes the distribution of clay, silt, and sand components within the soil. We also examined specific soil fractions, including the percentages of clay, silt, and sand by weight. Furthermore, soil organic carbon (SOC) content, a significant proxy for soil fertility, was considered^39^. these variables were sourced at a 500-meter spatial resolution with updates provided every five years, offering a granular and periodic evaluation of the soil’s physical and chemical characteristics. Such environmental data, categorized as periodic variables, is indispensable for accurately modeling the enduring impacts of soil conditions on agricultural productivity and sustainability (Table 4).

#### Agricultural modernization and geospatial data

Agricultural modernization data play an indispensable role in crop yield estimation^53^. This study selected the Degree of Fertilizer Consumption (DFC) and Degree of Comprehensive Mechanization (DCM) as key input variables for the crop yield model, sourced from agricultural management data such as chemical fertilizer consumption and comprehensive mechanization rates documented in county statistical yearbooks.

**Fig. 2:**
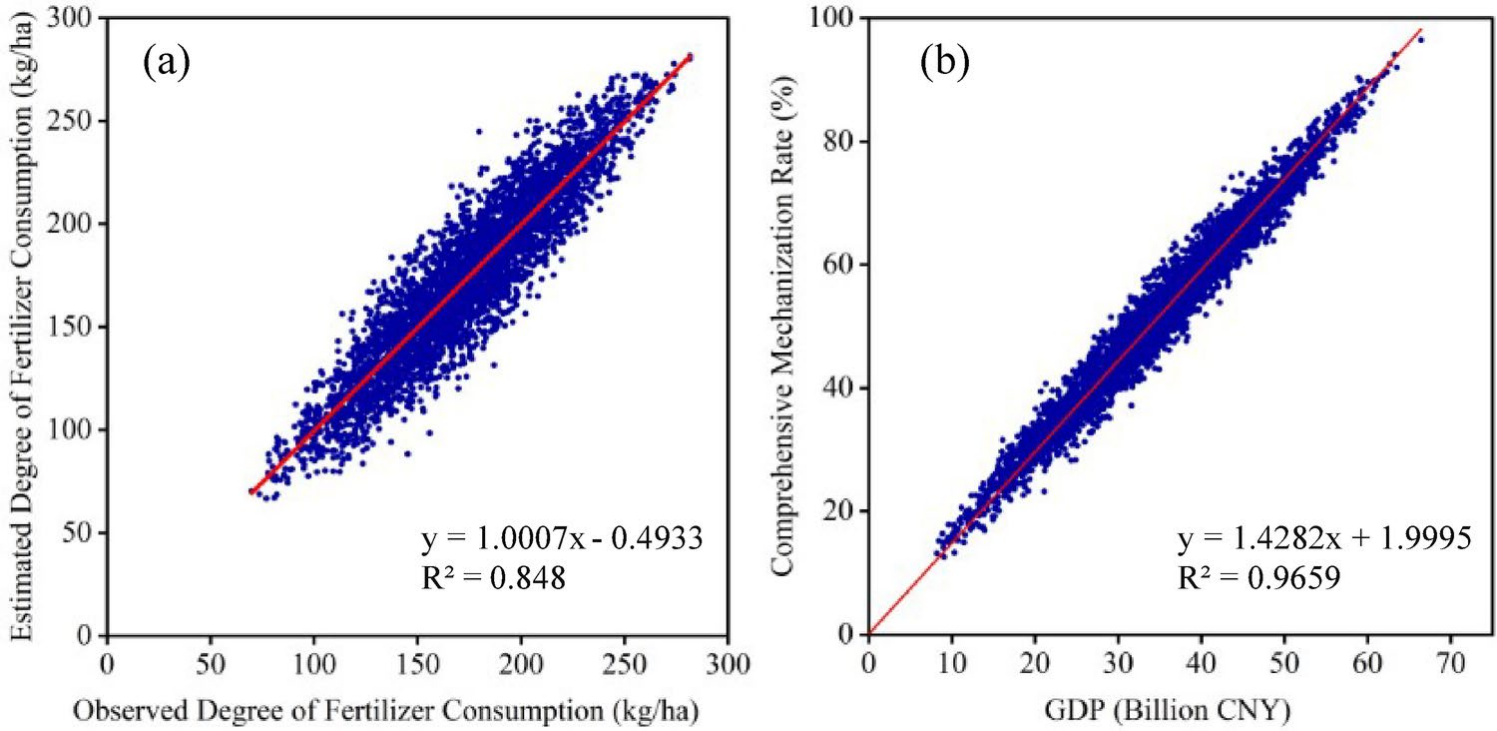
(**a**) Correlation between observed degree of fertilizer Consumption and estimated degree of fertilizer consumption (**b**) Correlation between GDP and comprehensive mechanization rates.

DFC was calculated by first extracting total fertilizer usage for all crops in each county from the statistical yearbooks. The proportion of maize planting area relative to the total crop area was used to estimate maize-specific fertilizer usage at the county level. To further refine this allocation, principal component analysis (PCA) was applied to the NDVI time series data, following the approach of Yang et al., who demonstrated the relationship between NDVI and fertilizer application^42^, and Sun et al., who identified significant correlations between the principal components of NDVI and both land quality and fertilizer usage^54^. The second principal component of the NDVI was employed as a proportional factor to spatially distribute maize-specific fertilizer usage. Fig.2a Correlation between observed and estimated Degree of Fertilizer Consumption (DFC), demonstrating a strong relationship that validates the accuracy of the estimation method and its applicability for DFC raster mapping, The resulting allocation was used to rasterize fertilizer data at a spatial resolution of 500 m, ensuring consistency with other model inputs.

DCM was derived from comprehensive mechanization rates provided in the statistical yearbooks, which include machine plowing, sowing, and harvesting data. For counties with missing mechanization data, maize’s share of the total crop area was used to proportionally adjust the values.

Mechanization rates were calculated as follows:

1 $$\:{R}_{p}=\frac{{A}_{p}}{{A}_{s}-{A}_{n}}\times\:100\%$$

2 $$\:{R}_{s}=\frac{{A}_{s}}{{A}_{t}}\times\:100\%$$

3 $$\:{R}_{h}=\frac{{A}_{h}}{{A}_{h}-{A}_{l}}\times\:100\%$$

The comprehensive mechanization rate $\:{R}_{c}$was computed as a weighted sum^55^:

4 $$\:{R}_{c}=0.4\cdot\:{R}_{p}+0.3\cdot\:{R}_{s}+0.3\cdot\:{R}_{h}$$

Based on prior studies demonstrating a strong correlation between regional mechanization levels and GDP, a model was developed using $\:{R}_{c}$from 2001 to 2021 and county-level GDP values derived from 1 km grid data^40^. Fig. 2b shows the fitting results of GDP 1 km grid extracted data and comprehensive mechanization rates, indicating a strong correlation between the two, which can serve as a basis for mapping DCM. Here, $\:{R}_{p},\:{R}_{s},and\:{R}_{h}$represent the rates of machine plowing, sowing, and harvesting, respectively, while$\:\:{A}_{p}{,\:A}_{s},\:{A}_{t}{,\:A}_{n},\:and\:{A}_{l}$ denote the areas for machine plowing, sowing, total sown area, no-till, and crop loss. This model generated DCM raster data for the entire study area, which was subsequently resampled to a 500 m spatial resolution to ensure compatibility with other datasets in the modeling pipeline.

**Fig. 3:**
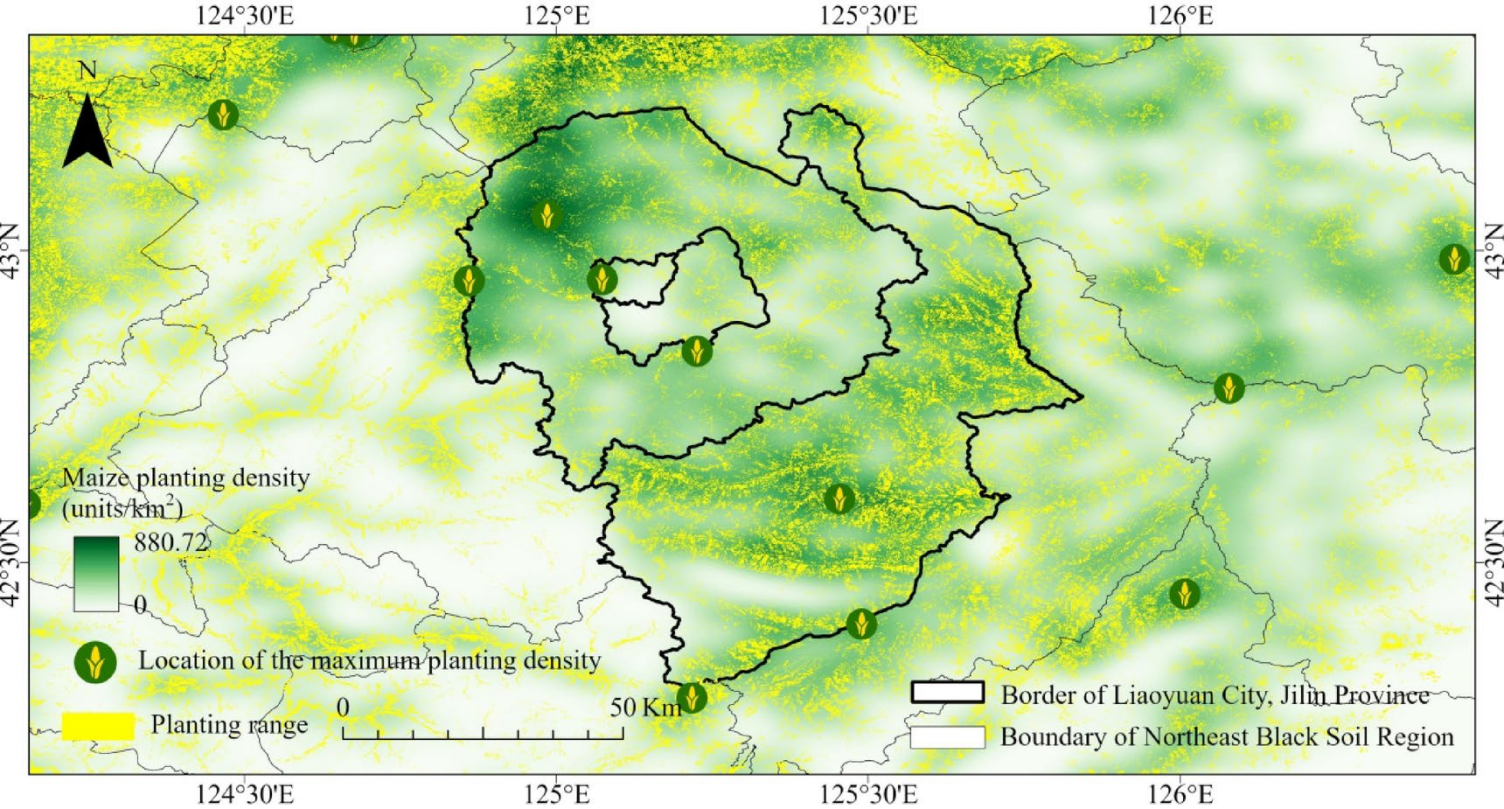
The location of the maximum planting density of maize in 2001 in the subordinate districts and counties of Liaoyuan City, Jilin Province. (Base map data adapted from GS(2020)4619, http://bzdt.ch.mnr.gov.cn/. Map generated using ArcGIS Pro 3.1.6, http://www.esri.com).

In previous crop yield estimation studies, the latitude and longitude of a county’s centroid were commonly used to represent the spatial characteristics of yield variation. However, this approach has limitations, as the centroid may not accurately reflect the actual distribution of maize cultivation within the county, particularly in regions where agricultural activities are highly concentrated or unevenly distributed. Therefore, we have replaced the centroid with the latitude and longitude of the highest maize planting density as a more precise representative location. Maize planting density was determined by analyzing the spatial distribution of planting areas across the study period. This was quantified by the count of maize field pixel units per square kilometer^36^, reflecting the concentration of cultivation. The resulting latitude and longitude coordinates were normalized and incorporated into the model as spatial tags, enabling the integration of precise spatial characteristics into the modeling. As shown in Fig. 3, in the case of Liaoyuan City, the location of the highest planting density often does not coincide with the county centroid, further demonstrating the effectiveness of this approach in more accurately identifying key agricultural production areas. These soil, geospatial, and agricultural modernization data are collectively categorized as periodic variables, capturing the temporal variability and impact of soil conditions, geospatial factors, and agricultural practices on crop productivity and sustainability (Table 4).

**Table 4:** Summary of periodic variables and processing.

Category	Variable	Description/Units	Range	Resolution	Chronology
Soil	Texture	Proportion of clay, silt, and sand	Integers 1–12	500 m	5 years
	Clay	Clay percentage by weight	0–88.18 (%)	500 m	5 years
	Silt	Silt percentage by weight	0–84.79 (%)	500 m	5 years
	Sand	Sand percentage by weight	0–90.04 (%)	500 m	5 years
	SOC	SOC percentage by weight	0–10 (%)	500 m	5 years
Geospatial	Longitude	Planting density center longitude	0–1	County level	1 years
	Latitude	Planting density center latitude	0–1	County level	1 years
AgriMD	Degree of fertilizer consumption (DFC)	Fertilizer consumption level percentage by weight in maize field	0–98.65 (%)	500 m	1 years
	Degree of cultivation mechanization (DCM)	Cultivation mechanization percentage by weight in maize field	69.8–281.6 (kg/ha)	500 m	1 years

### Attention-based hybrid CNNAtBiGRU model

**Fig. 4:**
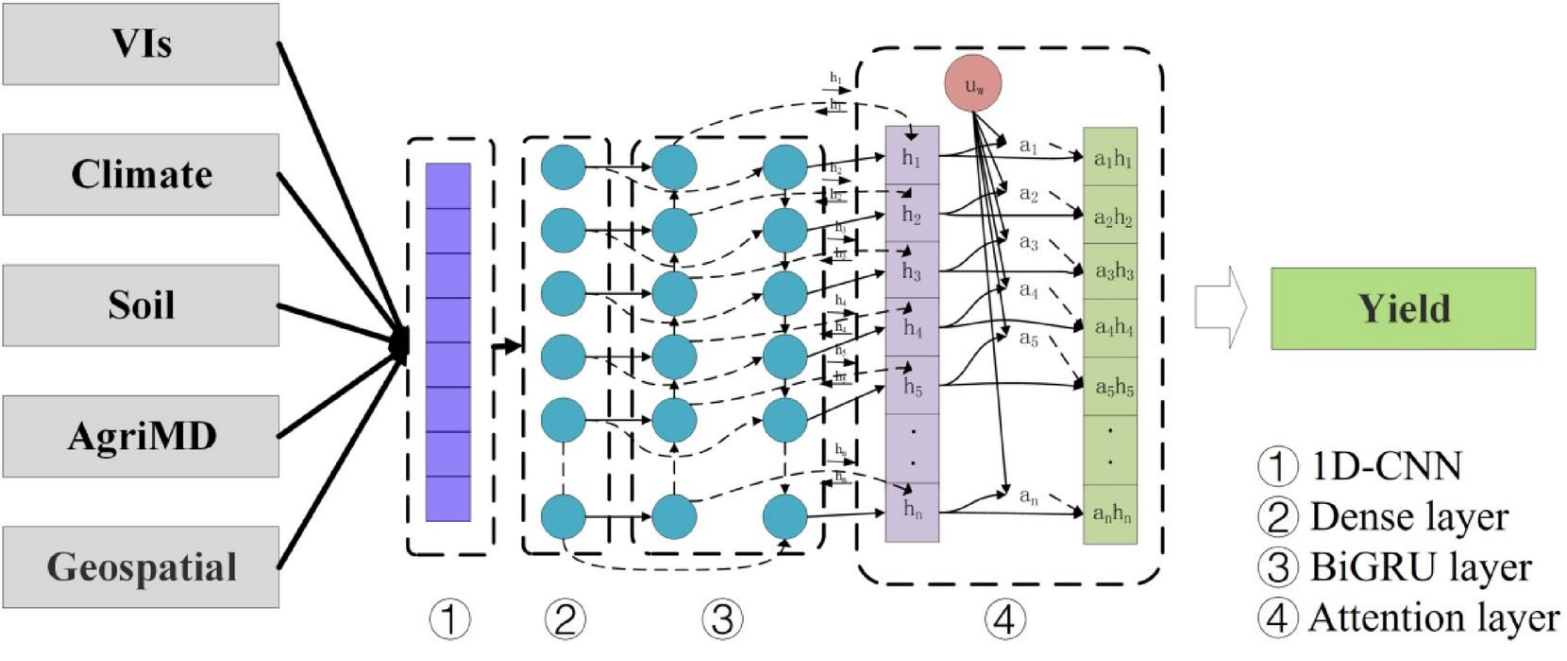
Attention-based hybrid CNNAtBiGRU model structure diagram.

The proposed CNNAtBiGRU deep learning framework is designed to optimize maize yield prediction by effectively integrating spatiotemporal data through a synergistic combination of modules. The framework, illustrated in Fig. 4, comprises an input layer (satellite data, climate, soil properties, agricultural modernization data, and geospatial information), a 1D-CNN layer, a BiGRU layer, and an attention layer. Each component plays a distinct role within the architecture: The CNN module extracts local spatial features from high-dimensional input data, capturing critical spatial patterns that are essential for understanding maize yield variability. The BiGRU module, leveraging its bidirectional structure, captures temporal dependencies within the time-series data, effectively modeling sequential relationships between variables. The attention mechanism, strategically placed after the BiGRU layer, identifies and emphasizes key features by assigning higher weights to the most relevant temporal states, ensuring that the model focuses on critical information for yield prediction.

This cohesive design allows CNNAtBiGRU to overcome limitations of traditional models by optimizing the integration of spatial and temporal features, enhancing predictive performance, and providing a more interpretable understanding of the key factors influencing maize yields in the black soil region.

#### Convolutional neural network

As a feedforward neural network, CNN has the capability to effectively handle time series prediction problems^56^. A CNN primarily comprises convolutional layers, pooling layers, and fully connected layers (Mustapha et al., 2024). The convolutional layer extracts one-dimensional features from continuous numerical data through the use of convolution filters and feature mapping. This feature extraction process is particularly valuable in identifying local spatiotemporal patterns from multisource input data, such as satellite imagery and climate data, which are critical for accurate yield predictions. In this model, multiple convolutional layers are sequentially applied to deepen the feature extraction process, thereby enhancing the robustness and discriminative power of the prediction task.

Each convolutional layer in the model contains a varying number of one-dimensional kernels of size (2 × 1), specifically 64, 32, and 16 for the respective layers. Following the convolution operation, the Rectified Linear Unit (ReLU) activation function is employed to introduce non-linearity into the model, which aids in mitigating overfitting issues. The cascading convolutional layers ensure that hierarchical feature representations are captured, providing the subsequent BiGRU layers with enriched and compact input features. The process of applying the ReLU activation function to 1D convolution layers is as follows:

5 $$\:{y}_{j}^{\left(k\right)}={ReLU}_{\text{1,2},3\in\:h}\left[\sum_{i=1}^{N\left(k-1\right)}Conv1D\left({x}_{i}^{\left(k-1\right)},{W}_{i,j}^{\left(k\right)}\right)+{b}_{j}^{\left(k\right)}\right]$$

In this context, $\:{\text{x}}_{\text{i}}^{\left(\text{k}-1\right)}$ denotes the$\:\:\text{i}$th feature map in the $\:\left(k-1\right)$th layer, while $\:{\:\text{y}}_{\text{j}}^{\text{k}}$.and the $\:j$th feature map in the kth layer is represented by Moreover, the trainable convolutional kernel is denoted as $\:{\text{W}}_{\text{i},\text{j}}^{\left(\text{k}\right)}$, and $\:{\text{N}}_{\left(\text{k}-1\right)}$ refers to the total number of feature maps in the $\:\left(k-1\right)$th layer. The 1D convolution operation, without zero-padding, is applied to $\:{\text{x}}_{\text{i}}^{\left(\text{k}-1\right)}$ and $\:{\text{W}}_{\text{i},\text{j}}^{\left(\text{k}\right)}$, leading to a reduction in feature map dimensions from the$\:\left(k-1\right)$th layer to the $\:k$th layer. Additionally, $\:{\text{b}}_{\text{j}}^{\left(\text{k}\right)}$ represents the bias associated with the $\:j$th feature map in the $\:k$th layer. The following is how the ReLU activation function operates:

6 $$\:ReLU\left({P}_{h}\right)=\text{m}\text{a}\text{x}\left(0,{P}_{h}\right)$$

where,

7 $$\:{P}_{h}=\sum_{i=1}^{N\left(k-1\right)}Conv1D\left({x}_{i}^{\left(k-1\right)},{W}_{i,j}^{\left(k\right)}\right)+{b}_{j}^{\left(k\right)}$$

After passing through all 1D convolutional layers, the obtained 16 feature maps with the size of (4 × 1) are then fed into a dense layer containing 16 nodes. This dense layer serves to further process and integrate the information from the feature maps. Subsequently, the output features from the dense layer are passed to the BiGRU model, which is designed to identify and extract valid and temporally coherent information from the feature maps. By combining convolutional layers with a dense layer containing 16 nodes, the extracted features are further refined to ensure that meaningful spatial and temporal patterns are passed to the subsequent BiGRU layers.

#### Gated recurrent unit

The gated recurrent unit (GRU), introduced by Cho^26^, is an evolution of the recurrent neural network (RNN) designed to address the problems of vanishing and exploding gradients typically associated with traditional RNNs. GRU streamlines the complexity of LSTM by unifying the forget and input gates into a single ‘update gate.’ This consolidation reduces the number of parameters within the model while preserving its capacity to capture long-term dependencies within time series data. Within the GRU framework, the transmission of information is regulated by two gates: the update gate $\:{z}_{t}$ and the reset gate $\:{r}_{t}$. The update gate modulates the extent to which the previous hidden state is propagated to the current state, and the reset gate dictates the extent to which past information is to be discarded. This design choice, compared to LSTM, offers computational efficiency while maintaining performance, making it more suitable for real-time agricultural predictions that require high throughput and accuracy.

The GRU model’s operations can be mathematically delineated as follows:

8 $$\:{z}_{t}=\:\sigma\:\left({W}_{z}{x}_{t}+{U}_{z}{h}_{t-1}+{b}_{z}\right)$$

9 $$\:{r}_{t}=\:\sigma\:\left({W}_{r}{x}_{t}+{U}_{r}{h}_{t-1}+{b}_{r}\right)$$

10 $$\:{\stackrel{\sim}{h}}_{t}=tanh\left({W}_{h}{x}_{t}+\:{r}_{t}\odot\:\left({U}_{h}{h}_{t-1}\right)+{b}_{t}\right)$$

11 $$\:{h}_{t}=\left(1-{z}_{t}\right)\odot{h}_{t-1}+{z}_{t}\odot\:{\stackrel{\sim}{h}}_{t}$$

Equations (8), (9), (10) and (11) correspond to the Update gate, Reset gate, Candidate hidden state, and Final hidden state in a GRU model. In these equations, $\:{x}_{t}$represents the input at time $\:t$, and $\:{h}_{t-1}$ is the hidden state from the previous time step. The weight matrices $\:{W}_{z},{W}_{r},{W}_{h}$ correspond to the update gate, reset gate, and candidate hidden state for the input $\:{x}_{t}$, while the matrices $\:{U}_{z},{U}_{r},{U}_{h},$represent the same gates for the hidden state$\:\:{h}_{t-1}$. The bias vectors $\:{b}_{z},{b}_{r}{,b}_{t},$ are also associated with these gates. The update gate $\:{z}_{t}$ controls how much of the previous hidden state is carried forward, and the reset gate $\:{r}_{t}$ determines how much of the past information is considered. The candidate hidden state $\:{\stackrel{\sim}{h}}_{t}$ is calculated at time step $\:t$, and the final hidden state $\:{h}_{t}$ is a combination of the previous hidden state and the newly calculated candidate state. The final output, $\:{h}_{t}$, encapsulates the necessary sequential information and is passed on to the next layers for additional processing.

The GRU’s ability to dynamically adapt the contribution of historical and current inputs is critical for capturing agricultural trends influenced by sequential climate and soil data. This functionality ensures that relevant temporal dependencies are preserved while discarding redundant or irrelevant information.

#### Bi-directional GRU

Unlike traditional unidirectional GRU models, the BiGRU architecture processes input sequences in both forward and backward directions, thereby capturing both past and future contexts for each time step^57^. This bidirectional approach is particularly advantageous for agricultural predictions, where crop yields are influenced by interactions between past climate conditions and future management practices.

The BiGRU operations can be mathematically expressed as:

12 $$\:\overrightarrow{{h}_{t}}=GRU\left({x}_{t},{\overrightarrow{h}}_{t-1}\right)$$

13 $$\:\overleftarrow{{h}_{t}}=GRU\left({x}_{t},{\overleftarrow{h}}_{t-1}\right)$$

14 $$\:{h}_{t}={\alpha\:}_{t}\overrightarrow{{h}_{t}}+{\beta\:}_{t}\overleftarrow{{h}_{t}}+{b}_{t}$$

In this model, $\:{x}_{t}$ represents the input at time step t.The forward and backward outputs at each time step are denoted by $\:\overrightarrow{{h}_{t}}$ and $\:\overleftarrow{{h}_{t}}$. The generated BiGRU features from the $\:i$th features in the $\:p$th layer is denoted as $\:{h}_{t}$, which retains information across both forward and backward directions. This bidirectional structure allows the model to integrate contextual information more effectively, ensuring that the predictions are informed by both historical patterns and potential future trends.

#### Attention mechanism

Drawing inspiration from human visual behavior, the Attention mechanism computes a probability distribution over input features, assigning higher weights to those most relevant for the prediction task^58^. This selective focus enhances the model’s interpretability by highlighting critical inputs, such as extreme climate events or significant soil characteristics, that have a disproportionate impact on maize yields. Within the attention mechanism, each BiGRU feature is assigned a weight $\:{a}_{n}$ with the corresponding feature vector $\:{h}_{n}^{\left[p\right]}$ being emphasized in relation to the output labels. The attention function is mathematically defined as follows:

15 $$\:{\mu\:}_{i}=tanh\left(Wd\times\:flatten\left({h}_{n}^{\left[p\right]}\right)+b\right)$$

16 $$\:{a}_{n}=\frac{\text{e}\text{x}\text{p}\left({\mu\:}_{i}{u}_{w}\right)}{{\sum}_{n}\text{e}\text{x}\text{p}\left({\mu\:}_{i}{u}_{w}\right)}$$

17 $$\:a{v}^{t}=\sum_{n}{a}_{n}{h}_{n}^{\left[p\right]}$$

Here, $\:{h}_{n}^{\left[p\right]}$ represents the feature vector obtained from the BiGRU layer, which is processed through a single-layer neural network (*p* = 1) to compute $\:{\mu\:}_{i}$ as a hidden representation of $\:{h}_{n}^{\left[p\right]}$. The weight matrix $\:Wd$ and bias vector $\:b$ re initialized during neural network training. The similarity between $\:{\mu\:}_{i}$ and $\:{u}_{w}$. helps determine the influence of important parameters. The softmax function is then used to generate a normalized weight $\:{a}_{n}$ for each input feature. The final attentive feature $\:a{v}^{t}$ is passed through a dense layer with a linear activation function for maize yield prediction, ensuring that the output $\:{F}_{pred}$ integrates all critical information:

18 $$\:{F}_{pred}=Linear\left(\sum_{j}{W}_{kj}\times\:a{v}_{j}^{t}+{b}_{k}\right)$$

In this equation, $\:{W}_{kj}$ and $\:{b}_{k}\:$denote the weight matrix and bias vector, respectively. A linear activation function is incorporated into the proposed model for the final yield prediction. By leveraging the attention mechanism, the model effectively focuses on significant temporal and spatial features, allowing for more robust and explainable yield predictions.

### Model evaluation

To comprehensively evaluate the CNNAtBiGRU model’s performance, we applied distinct modeling strategies to assess its stability and adaptability under varying conditions.

For the stability assessment, a ‘train-validate-test’ approach was utilized to evaluate CNNAtBiGRU, CNN-GRU^59^, GRU^26^, LightGBM^60^, and LASSO^61^ five models performance across years. Yield data from a single year, such as 2021, was designated as the test set, ensuring it was completely excluded from both training and validation phases. The remaining years from 2001 to 2020 were randomly partitioned into training and validation sets in an 80–20% ratio. This strategy ensured the independence of the test set, enabling rigorous evaluation of the model’s generalizability. To maintain reproducibility and minimize uncertainties caused by random data splits, fixed pseudo-random seeds were employed during training and validation. Specific years, such as 2001, 2014, and 2021, were selected to test the model’s robustness under diverse scenarios, including early-year predictions, extreme weather conditions, and recent-year data.

**Fig. 5:**
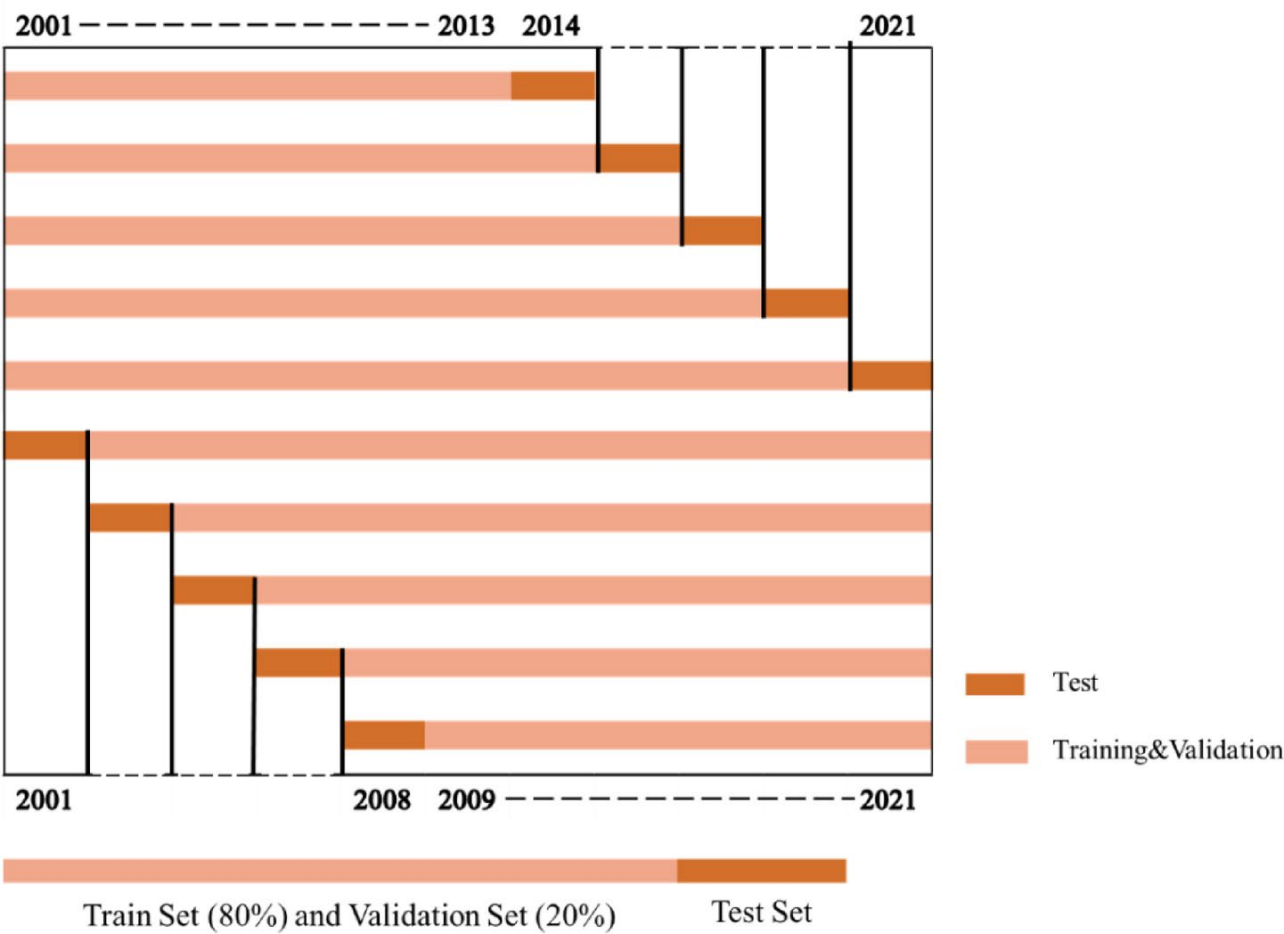
Data partitioning diagram (The dashed lines in the figure are indicative and not drawn to scale).

To explore the model’s adaptability and timeliness in capturing temporal dynamics, we adopted a strategy that sequentially modeled data forward and backward from a given year, simulating bidirectional temporal accumulation. In this setup, the years preceding or following the prediction year were randomly split into training and validation sets, while the prediction year itself served as the test set^62^. For instance, when 2014 was the test year, the training set included data from 2001 to 2013; similarly, for 2008, the training set comprised data from 2009 to 2021(Fig. 5).

In the context of early prediction, we gradually incorporated monthly-interval input data into the model to investigate the temporal progression of model performance across different growth stages, which is the in-season prediction. In other words, for any stage during the main growth period, the information from the beginning of the main growth period up to the current stage was utilized to predict maize yield. It was possible to determine when the best predictive performance could be achieved, that is, how accurately and early the proposed model could predict maize yields.

The overall model accuracy was measured using Root Mean Square Error (RMSE) and Coefficient of Determination (R²), defined as follows:

19 $$\:{R}^{2}=1-\frac{\sum{\left({y}_{i}-\widehat{{y}_{i}}\right)}^{2}}{\sum{\left({y}_{i}-\stackrel{-}{y}\right)}^{2}}$$

20 $$\:RMSE=\sqrt{\frac{\sum_{i=1}^{m}{\left({y}_{i}-\widehat{{y}_{i}}\right)}^{2}}{m}}\in\:\left[0,+\infty\:\right]$$

Where $\:{y}_{i}$ is the actual value, $\:\widehat{{y}_{i}}$ is the predicted value, $\:\widehat{{y}_{i}}$ is the mean of the actual values and $\:m$ is the total number of samples.

### Explanation of each prediction

We utilized Shapley additive explanations (SHAP) to quantify the contribution of individual features to the model’s predictions^63^. SHAP, proposed in 2017, employs a game-theoretic approach to interpret the outputs of any machine learning model. The SHAP value represents the difference between a sample’s prediction and the average prediction, thereby directly attributing each feature’s influence on the predicted yield. This method significantly enhances the interpretability of the model’s predictions.

Additionally, we employed permutation importance to assess the significance of features. This technique measures the impact on model performance—manifested as an increase in error—when the values of a specific feature are randomly shuffled, thus disrupting its correlation with the target variable. By observing the effect of permuting a feature on the model’s predictive accuracy, this approach provides insights into the relative importance of each feature. The combined use of SHAP values and permutation importance offers a robust framework for understanding feature significance and elucidating the mechanisms by which features influence the model’s predictions.

Both SHAP values and permutation importance analyses were integrated into the ‘train-validate-test’ strategy to ensure robust interpretation of feature contributions within this framework.

### Model parameter

Optimal hyperparameter tuning is crucial for training the proposed prediction model to achieve superior results. The Mean Squared Error (MSE), Mean Absolute Error (MAE), and RMSE are selected as evaluation metrics to assess the performance of the model after hyperparameter tuning. The model training and testing were conducted using Python version 3.10.12. Implementation was facilitated using the TensorFlow framework, version 2.8.2. For data preparation and evaluation, the Pandas library, version 1.5.3, and the Scikit-learn library, version 1.3.2, were employed, respectively.

The formula for RMSE is referenced from Eq. 13, MSE and MAE are defined as follows:

21 $$\:\text{MSE}=\frac{{\sum}_{i=1}^{m}{\left({y}_{i}-\widehat{{y}_{i}}\right)}^{2}}{m}$$

22 $$\:\text{MAE}=\frac{{\sum}_{i=1}^{m}\left|{y}_{i}-\widehat{{y}_{i}}\right|}{m}\in\:\left[0,+\infty\:\right]$$

Where $\:{y}_{i}$ is the actual value, $\:\widehat{{y}_{i}}$ is the predicted value, $\:\widehat{{y}_{i}}$ is the mean of the actual values and $\:m$ is the total number of samples.

**Fig. 6:**
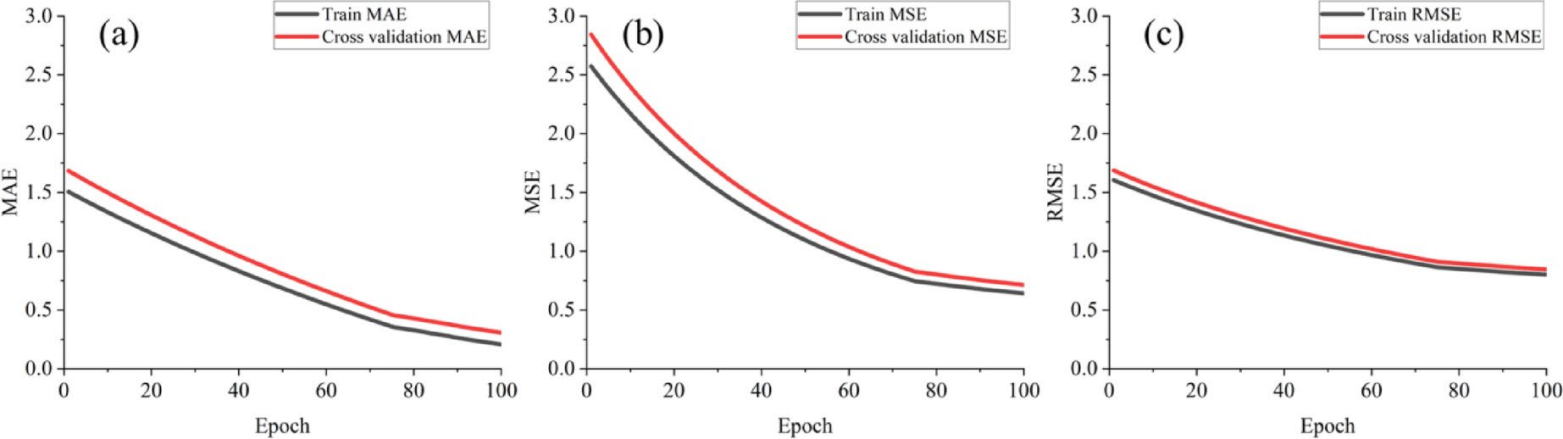
Performance evaluation on CNNAtBiGRU: (**a**) MAE, (**b**) MSE, and (**c**) RMSE.

Table 5 presents the performance metrics, showing that the CNNAtBiGRU model achieved near-optimal values through effective parameter tuning. Key hyperparameters, such as learning rate and batch size, were fine-tuned to enhance predictive accuracy. Figure 6 illustrates the training process, with MAE, MSE, and RMSE stabilizing at optimal levels after 95 epochs. Table 6 summarizes the optimal hyperparameters for the CNNAtBiGRU model. The model is compiled using the Adam optimizer, initialized with a learning rate of 0.01. Training is conducted over 95 epochs per fold.

**Table 5:** Performance evaluation matrices for maize yield prediction.

MAE	MSE	RMSE
0.358	0.746	0.864

**Table 6:** Hyperparameter settings for the proposed CNNAtBiGRU.

Hyper-parameters	Value
Initial learning rate	0.01
Epochs	95
Optimizer	Adam
Loss function	Mean Absolute Error Loss

## Results

### Performance of CNNAtBiGRU compared with that of other models

#### Temporal analysis of model performance across years

The temporal analysis of model performance from 2001 to 2021 highlights the superior stability and accuracy of the CNNAtBiGRU model compared to other methods. Using yield data as the test set and splitting the remaining data into training and validation sets at an 80:20 ratio, The obtained performance statistics are summarized in Tables 7 and 8, the evaluation demonstrates that CNNAtBiGRU consistently achieved the highest R² values, exceeding 0.8 in most years and peaking at 0.901 in 2001, significantly outperforming CNNGRU, GRU, LightGBM, and LASSO. Similarly, CNNAtBiGRU achieved the lowest RMSE values across nearly all years, generally below 1.0 t/ha, with a minimum of 0.892 t/ha in 2001. These results highlight CNNAtBiGRU’s robust capacity to capture temporal patterns, account for diverse agricultural conditions, and provide accurate and reliable yield predictions over time.

**Table 7:** Test R^2^ for CNNAtBiGRU, CNNGRU, GRU, LightGBM and LASSO models between 2001 and 2021.

Year	CNNAtBiGRU	CNNGRU	GRU	LightGBM	LASSO
2001	0.90 ± 0.01	0.69 ± 0.03	0.79 ± 0.01	0.82 ± 0.04	0.79 ± 0.02
2002	0.84 ± 0.02	0.52 ± 0.06	0.70 ± 0.02	0.76 ± 0.03	0.64 ± 0.10
2003	0.89 ± 0.01	0.68 ± 0.05	0.78 ± 0.01	0.80 ± 0.03	0.78 ± 0.03
2004	0.82 ± 0.02	0.43 ± 0.02	0.62 ± 0.03	0.62 ± 0.03	0.56 ± 0.16
2005	0.89 ± 0.01	0.66 ± 0.06	0.75 ± 0.02	0.78 ± 0.02	0.76 ± 0.02
2006	0.85 ± 0.00	0.56 ± 0.01	0.70 ± 0.02	0.76 ± 0.03	0.68 ± 0.04
2007	0.80 ± 0.01	0.41 ± 0.10	0.58 ± 0.02	0.52 ± 0.04	0.55 ± 0.10
2008	0.83 ± 0.02	0.45 ± 0.12	0.66 ± 0.03	0.65 ± 0.06	0.61 ± 0.06
2009	0.88 ± 0.01	0.66 ± 0.04	0.74 ± 0.01	0.78 ± 0.00	0.74 ± 0.04
2010	0.85 ± 0.01	0.57 ± 0.03	0.70 ± 0.04	0.76 ± 0.04	0.68 ± 0.09
2011	0.87 ± 0.01	0.64 ± 0.06	0.73 ± 0.02	0.77 ± 0.03	0.74 ± 0.02
2012	0.86 ± 0.01	0.62 ± 0.06	0.72 ± 0.01	0.77 ± 0.03	0.73 ± 0.05
2013	0.86 ± 0.04	0.63 ± 0.04	0.73 ± 0.01	0.77 ± 0.03	0.73 ± 0.09
2014	0.83 ± 0.01	0.44 ± 0.03	0.62 ± 0.03	0.62 ± 0.12	0.57 ± 0.16
2015	0.89 ± 0.02	0.68 ± 0.04	0.76 ± 0.01	0.78 ± 0.03	0.77 ± 0.04
2016	0.82 ± 0.01	0.42 ± 0.02	0.61 ± 0.10	0.57 ± 0.06	0.56 ± 0.04
2017	0.85 ± 0.02	0.59 ± 0.07	0.72 ± 0.01	0.77 ± 0.01	0.69 ± 0.06
2018	0.85 ± 0.01	0.58 ± 0.02	0.71 ± 0.00	0.76 ± 0.08	0.69 ± 0.02
2019	0.83 ± 0.01	0.45 ± 0.07	0.63 ± 0.01	0.65 ± 0.04	0.59 ± 0.04
2020	0.84 ± 0.01	0.50 ± 0.06	0.69 ± 0.02	0.67 ± 0.03	0.63 ± 0.01
2021	0.89 ± 0.06	0.73 ± 0.01	0.79 ± 0.02	0.84 ± 0.03	0.82 ± 0.05

**Table 8:** Test RMSE (t/ha) for the CNNAtBiGRU, CNNGRU, GRU, LightGBM and LASSO models between 2001 and 2021.

Year	CNNAtBiGRU	CNNGRU	GRU	LightGBM	LASSO
2001	0.893 ± 0.01	1.153 ± 0.026	1.242 ± 0.03	1.063 ± 0.015	1.174 ± 0.04
2002	0.9314 ± 0.005	1.444 ± 0.012	1.339 ± 0.013	1.285 ± 0.01	1.371 ± 0.049
2003	0.924 ± 0.006	1.327 ± 0.013	1.265 ± 0.003	1.109 ± 0.014	1.179 ± 0.01
2004	0.974 ± 0.001	1.627 ± 0.05	1.376 ± 0.008	1.445 ± 0.03	1.473 ± 0.05
2005	0.9254 ± 0.003	1.354 ± 0.081	1.277 ± 0.001	1.128 ± 0.021	1.285 ± 0.009
2006	0.9304 ± 0.002	1.442 ± 0.02	1.329 ± 0.007	1.282 ± 0.04	1.365 ± 0.013
2007	1.0423 ± 0.006	1.641 ± 0.017	1.413 ± 0.039	1.473 ± 0.04	1.482 ± 0.05
2008	0.9389 ± 0.006	1.469 ± 0.025	1.344 ± 0.012	1.342 ± 0.02	1.391 ± 0.04
2009	0.9263 ± 0.008	1.358 ± 0.024	1.282 ± 0.005	1.129 ± 0.067	1.289 ± 0.039
2010	0.9302 ± 0.007	1.409 ± 0.006	1.319 ± 0.001	1.236 ± 0.021	1.358 ± 0.064
2011	0.9272 ± 0.004	1.363 ± 0.021	1.292 ± 0.007	1.143 ± 0.016	1.3 ± 0.024
2012	0.9281 ± 0.011	1.377 ± 0.085	1.294 ± 0.012	1.166 ± 0.004	1.332 ± 0.025
2013	0.9281 ± 0.007	1.376 ± 0.02	1.293 ± 0.005	1.149 ± 0.021	1.307 ± 0.005
2014	0.9716 ± 0.012	1.516 ± 0.01	1.367 ± 0.01	1.398 ± 0.029	1.457 ± 0.02
2015	0.9248 ± 0.006	1.333 ± 0.006	1.275 ± 0.003	1.124 ± 0.011	1.18 ± 0.021
2016	0.982 ± 0.01	1.635 ± 0.037	1.381 ± 0.019	1.471 ± 0.015	1.479 ± 0.033
2017	0.9283 ± 0.008	1.378 ± 0.013	1.299 ± 0.018	1.174 ± 0.03	1.339 ± 0.058
2018	0.9297 ± 0.002	1.385 ± 0.013	1.308 ± 0.001	1.192 ± 0.003	1.349 ± 0.022
2019	0.9398 ± 0.026	1.479 ± 0.026	1.352 ± 0.03	1.369 ± 0.021	1.395 ± 0.017
2020	0.9369 ± 0.005	1.456 ± 0.049	1.343 ± 0.005	1.298 ± 0.014	1.377 ± 0.071
2021	0.9083 ± 0.009	1.143 ± 0.02	1.236 ± 0.02	1.038 ± 0.018	1.034 ± 0.012

#### Spatial analysis of model performance in representative years

**Fig. 7:**
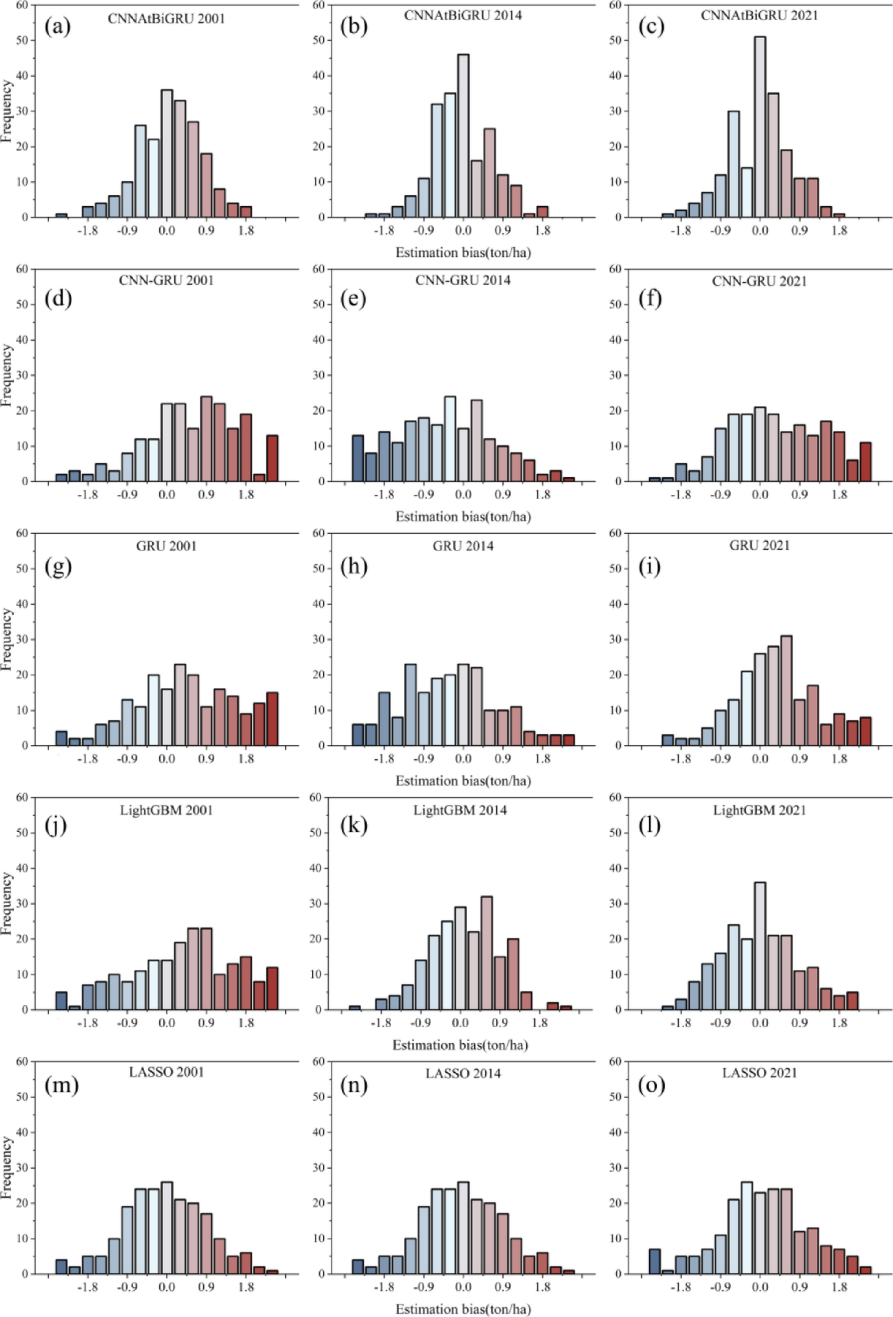
Frequency histogram of county-level maize errors for (**a**-**c**) CNNAtBiGRU, (**d**-**f**) CNNGRU, (**g**-**i**) GRU, (**j**-**l**) LightGBM, and (**m**-**o**) LASSO models for the representative years 2001, 2014, and 2021.

This study selected 2001, 2014, and 2021 as representative years for maize yield prediction in Northeast China. These years were chosen to capture early conditions (2001), an extreme drought event (2014), and the most recent data (2021). Figure 7 presents frequency histograms of county-level maize yield errors across five models for these years, showing the number of counties with over- or underestimation. The CNNAtBiGRU model demonstrated the fewest counties with significant overestimation or underestimation (defined as estimation bias exceeding ± 0.9 tons per hectare), outperforming CNN-GRU, GRU, LightGBM, and LASSO, particularly under extreme climatic conditions.

**Fig. 8:**
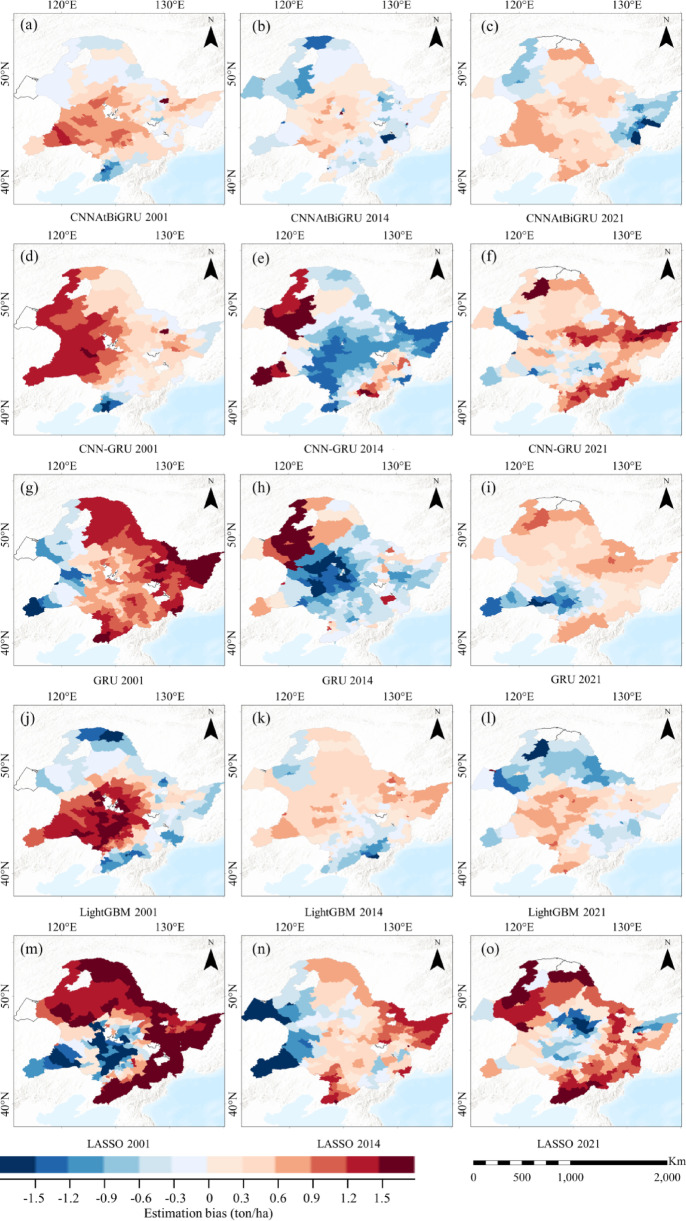
Spatial comparison of estimation bias accuracy (ton/ha) among (**a**-**c**) CNNAtBiGRU, (**d**-**f**) CNNGRU, (**g**-**i**) GRU, (**j**-**l**) LightGBM, and (**m**-**o**) LASSO models for the representative years 2001, 2014, and 2021. (Base map data adapted from GS(2020)4619, http://bzdt.ch.mnr.gov.cn/. Map generated using ArcGIS Pro 3.1.6, http://www.esri.com).

Spatial analysis of estimation bias in Figure 8. further highlights the accuracy of the CNNAtBiGRU model in different years and regions. In 2001, CNNAtBiGRU overestimated yields in 15 counties and underestimated in 14 counties, primarily located in the southwestern grassland-sandy areas of the study region (Fig. 8a). In contrast, other models exhibited larger errors; for example, LightGBM significantly overestimated yields in 58 counties (Fig. 8j) and LASSO underestimated yields in 26 counties (Fig. 8m), respectively, mainly in the central maize-producing region. In 2014, an extreme drought year, CNNAtBiGRU’s errors were limited to 13 overestimations and 11 underestimations, mostly in the northern forested areas of the study region (Fig. 8b). By comparison, CNN-GRU and GRU substantially underestimated yields in the central maize-producing region, affecting 63 and 58 counties, respectively (Fig. 8e, h). LightGBM and LASSO overestimated yields in 28 and 24 counties, respectively, with significant deviations also concentrated in the central maize belt (Fig. 8k, n). By 2021, CNNAtBiGRU maintained a balanced error distribution with 15 overestimations and 14 underestimations (29 counties in total), predominantly in the western wetland-lake areas of the study region (Fig. 8c). Meanwhile, CNN-GRU, GRU, LightGBM, and LASSO recorded substantial estimation bias exceeding ± 0.9 tons per hectare in 78, 59, 52, and 60 counties, respectively, with significant deviations concentrated in the central maize-producing region (Fig. 8f, i, l, o). Notably, CNNAtBiGRU consistently demonstrated lower estimation errors across the early year, extreme climate year, and recent year, with the error-prone areas primarily located outside the main maize-producing regions. These results underline the superior accuracy and robustness of CNNAtBiGRU in managing spatial heterogeneity and predicting maize yields under diverse climatic and temporal conditions.

### Timeliness of CNNAtBiGRU for yield prediction

#### Bidirectional prediction capability for maize yield

To evaluate the temporal adaptability of the model, we implemented a two-way validation process. First, data from earlier years were used as the training set to predict maize yields for 2014–2021, assessing the model’s performance in more recent years (Table 9). Conversely, data from recent years were employed as the training set to verify the model’s accuracy in forecasting maize yields for 2001–2008 (Table 10). Independent of the specific years, focusing on the size of the training dataset reveals that models trained on larger datasets generally exhibit better performance. Specifically, CNNAtBiGRU models trained on 20 years of observational data explained nearly 90% of the yield variation, significantly outperforming models trained on 13 years of data, which achieved R² values of 0.653 and 0.582, respectively. Correspondingly, RMSE values declined from approximately 1.4 tons/ha with 13 years of training data to around 0.90 tons/ha with 20 years. Regardless of whether recent years were used to validate earlier years or vice versa, the model exhibited consistent accuracy between the validation periods. These results demonstrate that CNNAtBiGRU effectively reduces the uncertainty associated with using historical data to estimate future maize yields and using recent records to predict historical yields.

**Table 9:** RMSE (ton/ha) and R^2^ of CNNAtBiGRU for recent test years.

Training set	R^2^ and RMSE (ton/ha) in test year							
(n = sample size)	2014	2015	2016	2017	2018	2019	2020	2021
2001-2013 (n=2613)	0.653 (1.3939)	0.683 (1.3252)	0.663 (1.36448)	0.623 (1.45071)	0.663 (1.36722)	0.573 (1.55146)	0.553 (1.59187)	0.653 (1.38594)
2001-2014 (n=2814)		0.754 (1.18256)	0.784 (1.12545)	0.754 (1.18412)	0.744 (1.20825)	0.734 (1.2235)	0.724 (1.24399)	0.714 (1.26279)
2001-2015 (n=3015)			0.804 (1.08365)	0.734 (1.22859)	0.794 (1.1054)	0.754 (1.18382)	0.734 (1.23092)	0.744 (1.2049)
2001-2016 (n=3216)				0.744 (1.20941)	0.754 (1.18971)	0.754 (1.18353)	0.794 (1.10966)	0.764 (1.1692)
2001-2017 (n=3417)					0.784 (1.13051)	0.814 (1.06218)	0.784 (1.12422)	0.804 (1.08317)
2001-2018 (n=3618)						0.834 (1.02446)	0.854 (0.98359)	0.824 (1.04272)
2001-2019 (n=3819)							0.879 (0.93946)	0.87 (0.95819)
2001-2020 (n=4019)								0.896 (0.90833)

**Table 10:** RMSE (ton/ha) and R^2^ of CNNAtBiGRU for early test years.

Training set	R^2^ and RMSE (ton/ha) in test year							
(n = sample size)	2008	2007	2006	2005	2004	2003	2002	2001
2009-2021 (n=2612)	0.582 (1.4299)	0.664 (1.36746)	0.612 (1.47211)	0.603 (1.4892)	0.642 (1.41259)	0.662 (1.36611)	0.674 (1.34226)	0.664 (1.37043)
2008-2021 (n=2813)		0.705 (1.28764)	0.772 (1.15103)	0.745 (1.201)	0.703 (1.28454)	0.697 (1.29746)	0.657 (1.38595)	0.709 (1.27333)
2007-2021 (n=3014)			0.831 (1.0355)	0.666 (1.36075)	0.822 (1.05301)	0.7 (1.29376)	0.717 (1.262)	0.763 (1.17064)
2006-2021 (n=3215)				0.696 (1.30357)	0.757 (1.18584)	0.711 (1.26853)	0.761 (1.17378)	0.701 (1.2972)
2005-2021 (n=3416)					0.727 (1.23749)	0.782 (1.13412)	0.78 (1.13614)	0.816 (1.06763)
2004-2021 (n=3617)						0.809 (1.07271)	0.845 (1.00727)	0.848 (0.99796)
2003-2021 (n=3818)							0.833 (1.03285)	0.889 (0.92119)
2002-2021 (n=4019)								0.901 (0.89338)

#### Early prediction of maize yield

**Fig. 9:**
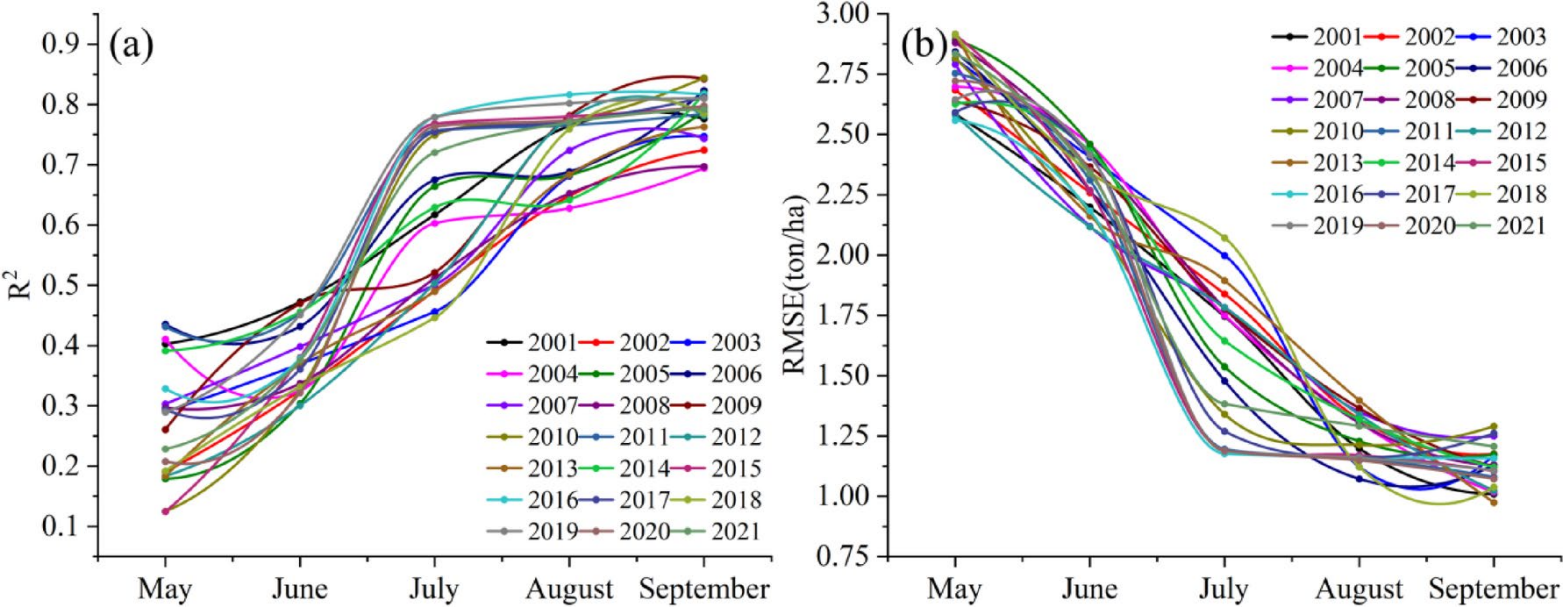
Verify test (**a**) R^2^ and (**b**) RMSE from maize by month stage from 2001 to 2021.

Figure 9 illustrates the results of in-season maize yield predictions from May to September using the proposed methodology. Figure 9 (a) shows the temporal changes in R² accuracy across different years. Notably, R² accuracy improved significantly between July and August for most years, reaching its peak in September. Figure 9(b) presents the temporal changes in RMSE (root mean square error) across different years. Similarly, RMSE decreased markedly between July and August, reaching its lowest value in September. For certain years, including 2010, 2011, 2015, 2016, 2017, 2019, and 2020, relatively high prediction accuracy was achieved as early as July. However, with the accumulation of maize growth information, most years exhibited significantly higher prediction accuracy in August. These findings demonstrate that the CNNAtBiGRU model effectively captures growth-related information as the season progresses, enabling accurate yield predictions 1–2 months in advance.

### Ablation experiments

#### Ablation experiment among modules

We carried out ablation experiments on different modules of CNNAtBiGRU, a model proposed in this study and applied to maize yield estimation. The ablation experiment results show that removing different model components impacts maize yield prediction. The complete model achieves the highest R^2^of 0.896 and the lowest RMSE of 908 kg/ha, indicating optimal performance. When without the CNN, the model’s R^2^drops to 0.809 and RMSE rises to 1080 kg/ha. Removing one GRU layer results in an R^2^of 0.801 and RMSE of 1103 kg/ha, showing a moderate impact. Removing the attention layer reduces R^2^ to 0.779 and increases RMSE to 1113 kg/ha, highlighting its critical importance. Overall, the attention layer has the most significant impact, followed by the GRU layer and the CNN (Table 11).

**Table 11:** Ablation experiment among model components.

Methods	*R* ^2^	RMSE (kg/ha)
Our Method	0.896 ± 0.010	908 ± 20
W/O CNN	0.809 ± 0.006	1080 ± 14
W/O 1 GRU Layer	0.801 ± 0.008	1103 ± 17
W/O Attention layer	0.779 ± 0.012	1133 ± 19

#### Ablation experiment among different combinations of datasets

We divided the input data into three types of time scales. The first type is the monthly data that changes every month (such as remote sensing VI and meteorological data), the second type is the data that changes every year (such as DCM), and the third type is the input data that changes every five years (such as soil information). Ablation experiments were carried out on these three types of data. The results show that the removal of data at different time scales has a significant effect on the model’s maize yield prediction. Specifically, excluding one month of data decreases the R²to 0.729 and increases the RMSE to 1233 kg/ha, indicating the critical importance of short-term data for model accuracy. Removing one year of data, which includes annual measurements of anthropogenic factors such as the coordinates of maize planting density centers at the county level, DCM and DFC, results in an R²of 0.785 and an RMSE of 1098 kg/ha, showing a smaller impact on model performance. Removing data from a five-year period yields an R²of 0.807 and an RMSE of 1065 kg/ha, demonstrating the least impact. The model performs best with the complete dataset, achieving an R^2^ of 0.896 and an RMSE of 908 kg/ha, underscoring the importance of utilizing all available data to maximize predictive accuracy (Table 12).

**Table 12:** Ablation experiment among different data.

Methods	*R* ^2^	RMSE (kg/ha)
W/O 1 month data	0.729 ± 0.006	1233 ± 17
W/O 1 year data	0.785 ± 0.007	1098 ± 20
W/O 5 years data	0.807 ± 0.008	1065 ± 16
Our Method	0.896 ± 0.010	908 ± 20

### Model interpretation

**Fig. 10:**
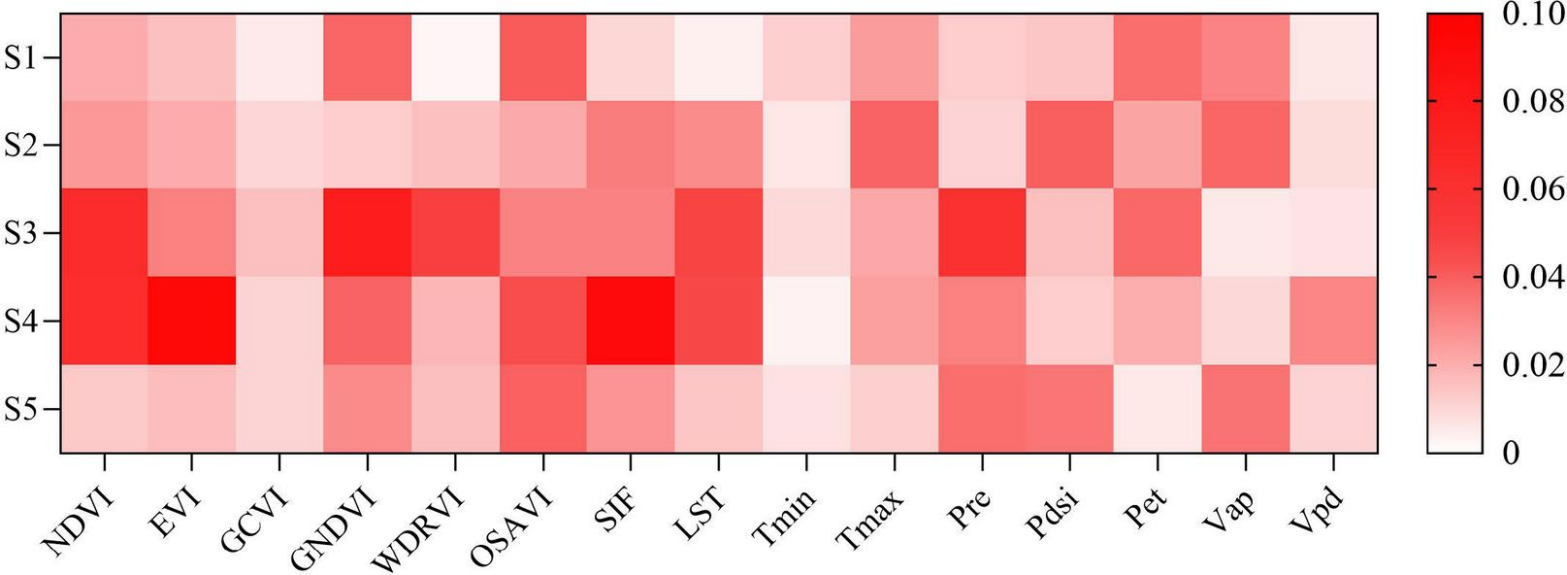
Permutation importance of dynamic feature.

To facilitate clear naming and labeling in the figures, we defined May through September as S1 to S5, respectively. We further analyzed the importance of input variables in the model by means of permutation importance (Fig. 10). Vegetation and surface indices derived from satellite imagery, such as EVI, GNDVI, NDVI, OSAVI, and LST, contribute more significantly to yield predictions than environmental climate data. Among the top 15 variables, EVI has the highest value and plays the most critical role in yield forecasting. In environmental climate data, Pre, Pdsi and Tmax are important variables for yield prediction, but their significant contributions are limited to specific periods rather than the entire growing season, resulting in a smaller overall impact compared to vegetation indices. Overall, vegetation indices hold a greater weight among the key indicators involved in yield prediction, as indicated by the larger number of dark red squares in Fig. 10, typically appearing in the third, and fourth periods. In contrast, the contribution of meteorological data across different periods shows inconsistency. Notably, precipitation during the third period is relatively important.

**Fig. 11:**
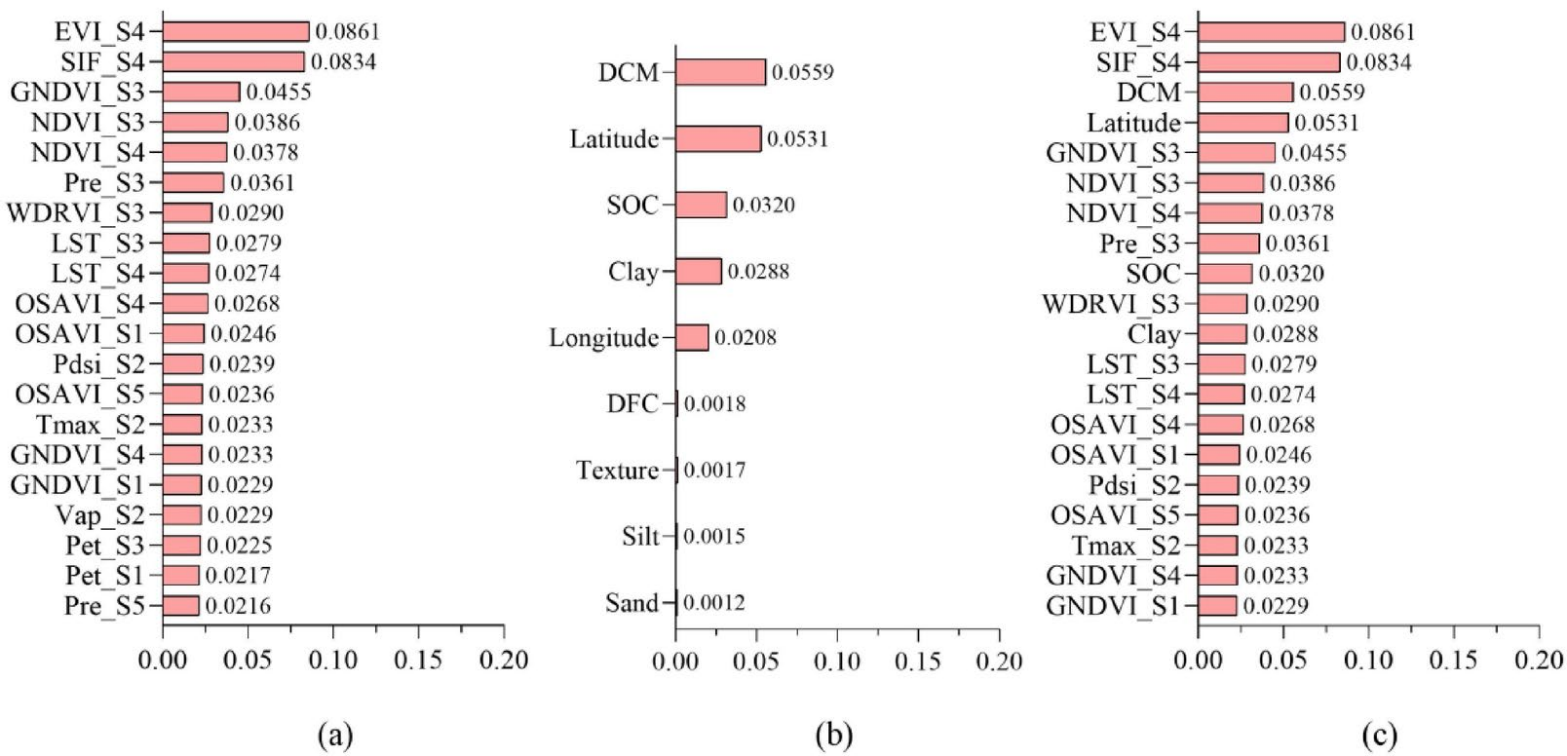
Permutation importance value: (**a**) dynamic variables, (**b**) periodic variables (**c**) top 20 variables among all variables.

We refer to variables with a usage cycle of less than one year as quarterly variables, such as NDVI, which is updated monthly. Variables with a usage cycle of one year or more are referred to as long-term variables, such as soil variables and latitude/longitude variables. As shown in Fig. 11a, the 20 most important quarterly variables indicate that the EVI in the fourth period has the highest importance at 8.61%, followed by SIF in the same period. This is followed by GNDVI and NDVI in the third period. The environmental climate data Pre from the third period ranks sixth, with an importance of 3.61%. Among these quarterly variables, the top ten are all from the third and fourth periods. The importance of long-term variables is shown in Fig. 11b. The DCM and geographical latitude information are more important than soil characteristics. Additionally, they still hold a prominent position in the overall ranking (Fig. 11c). CCF, SOC and other soil variables such as Clay also have some impact on maize yield prediction, while sand and silt content in soil types contribute less to maize yield prediction. Comparing quarterly variables and long-term variables, we find that 16 of the top 20 variables are quarterly variables and 4 are long-term variables (ranking 3rd, 4 th, 9 th and 11 th). The Satellite data (NDVI, EVI, GNDVI, WDRVI, OSAVI, SIF, LST) accounted for 13 variables, and environmental factors (Pre, Pdsi, Tmax, SOC, Clay) accounted for 5 variables. Among the 20 variables, 11 are derived from the third and fourth periods, which predominantly represent the mid-growth phase. This underscores the critical role of factors such as nutrient availability and early growth stages in accurately predicting yields.

**Fig. 12:**
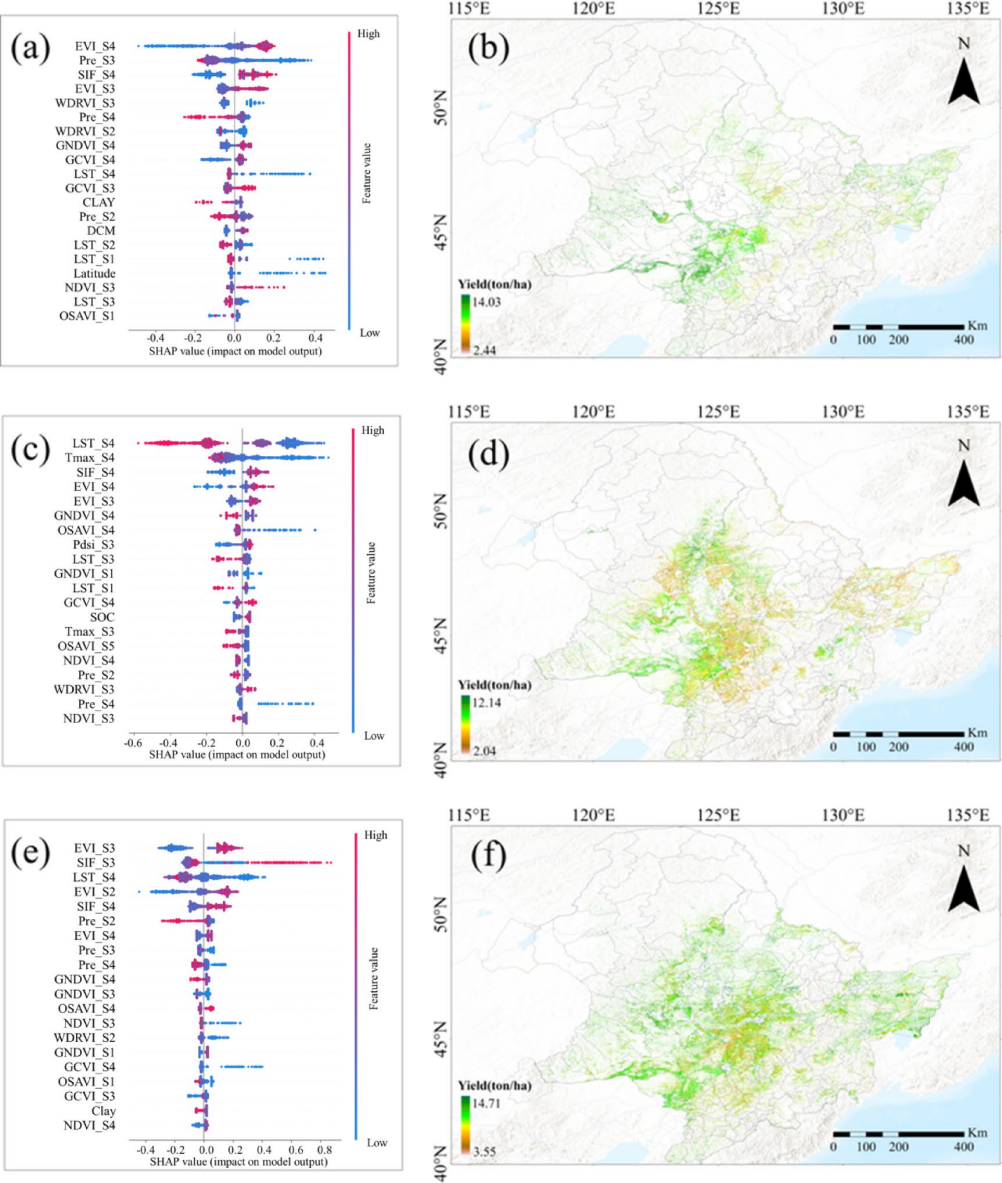
SHAP values illustrating feature importance for maize yield prediction in (**a**) an earlier year (2001), (**c**) a drought year (2014), and (**e**) a normal weather year (2021). The corresponding maps (**b**), (**d**), and (**f**) show the spatial distribution of maize yield predictions for the respective years. (Base map data adapted from GS(2020)4619, http://bzdt.ch.mnr.gov.cn/. Map generated using ArcGIS Pro 3.1.6, http://www.esri.com).

The SHAP analysis was conducted to evaluate the importance of the top 20 input variables for maize yield prediction during extreme weather years, specifically within the framework of the attention mechanism (Fig. 12). This analysis identifies the contribution of each variable to the model’s predictions, providing valuable insights into their relative significance under varying climatic conditions. SHAP values indicate the impact of each variable on the final yield predictions: higher absolute SHAP values denote greater importance, while the sign (positive or negative) reflects whether the variable had a positive or negative influence on the yield.

Three distinct years were selected for this analysis: 2001 (Fig. 12a), representing an earlier period, 2014 (Fig. 12c), marked by extreme climate events, and 2021 (Fig. 12e), considered a normal year for comparison. The SHAP value plots (Fig. 12a, c and e) highlight the most influential variables during each year, while the yield distribution maps (Fig. 12b, d and f) visualize the spatial patterns of maize yield predictions in Northeast China under varying climatic conditions. This combination allows for a comprehensive evaluation of how input variables behaved under different weather conditions, both in terms of their individual contributions and their effects on spatial yield distribution.

In 2001, a year with relatively high precipitation, precipitation-related variables were notably important but exhibited negative SHAP values. This suggests that excessive rainfall likely had adverse effects on maize yield, possibly due to waterlogging or other stressors that hindered crop growth. The yield distribution map for 2001 (Fig. 12b) reflects these adverse effects, with certain regions showing lower predicted yields.

In 2014, an extreme climate year, elevated temperatures were a major concern. Many counties in Northeast China experienced significantly high land surface temperatures (LST), which had a pronounced negative impact on maize yield, as reflected by the negative SHAP values for LST. The corresponding yield distribution map (Fig. 12d) shows a notable decline in yields in regions experiencing extreme heat. This indicates that extreme heat contributed to yield reductions, a well-known phenomenon where high temperatures cause heat stress, reduce pollination success, and lead to lower productivity.

In 2021, a normal year, variables like EVI and SIF had positive SHAP values, highlighting the importance of vegetation health and photosynthetic activity in predicting maize yield. EVI and SIF are proxies for crop health and growth vigor; their positive contribution suggests that healthier crops, as indicated by higher EVI and SIF values, were associated with higher yields during the growing season. The yield distribution map for 2021 (Fig. 12f) reflects this, with higher predicted yields in regions with healthier vegetation conditions.

The SHAP analysis thus reinforces the importance of key environmental indicators such as EVI, SIF, precipitation, and LST in predicting crop yields. The varying impact of these variables across different climatic years illustrates how prediction models dynamically adapt to changing weather patterns, with temperature and precipitation showing negative effects during extreme conditions, while indicators of crop health maintain their positive influence in normal years. Notably, variables from the S3 and S4 growth stages accounted for 12, 15, and 15 of the top 20 most important variables in 2001, 2014, and 2021, respectively. This finding aligns with Fig. 9, emphasizing the potential for early yield predictions up to 1–2 months before harvest. The analysis underscores the value of SHAP in interpreting complex models and understanding variable interactions under diverse environmental stresses, as demonstrated through feature importance and spatial yield distribution.

## Discussion

### Advantages of CNNAtBiGRU

The CNNAtBiGRU model’s enhanced performance is primarily due to the attention mechanism, which optimizes both CNN and BiGRU components. This mechanism prioritizes the most relevant spatial and temporal features, improving the model’s ability to capture complex patterns in maize yield prediction^64^. The CNN layer extracts key spatial features from environmental inputs, such as soil and climate factors, while the attention mechanism highlights influential regions, addressing spatial variability challenges often faced by traditional models^65^.

On the temporal side, BiGRU captures past and future dependencies in time series data, with the attention mechanism focusing on critical time steps^66^. This proves advantageous in volatile environments, such as extreme climate years (e.g., 2014). CNNAtBiGRU excels in early yield prediction, leveraging past and present data to predict future yields and estimate earlier yields (e.g., the 1990 s)^67^. This bidirectional capability enables early forecasting with high accuracy, even with incomplete historical data. The model’s performance improves with year-by-year and month-by-month data aggregation, consistent with studies highlighting the value of continuous data flow for refining predictions^68–70^. For instance, maize yields can be reliably predicted up to two months before harvest, with the 2021 forecast slightly improving earlier timelines.

In comparison to traditional models like LASSO and LightGBM, which often struggle with nonlinear relationships and complex temporal patterns, CNNAtBiGRU excels due to its dynamic weighting of spatial and temporal features^61^. This adaptability also makes it more robust in handling incomplete or missing data, a crucial aspect for early and long-term forecasting.

### Diversity of model variables

This study underscores the significance of integrating multi-source phenological data, encompassing meteorological, soil, topographical, and spectral index information, for enhancing county-level maize yield prediction. Year-by-year and month-by-month data analysis shows that model performance improves with data accumulation^71^. enabling reliable yield predictions up to two months before harvest, slightly earlier than previous studies^72,73^.

While traditional approaches rely on natural environmental data, incorporating agricultural management factors such as mechanized acreage and fertilizer application rates provides a more comprehensive understanding of yield dynamics^74^. For instance, mechanized acreage reflects farming efficiency, while fertilizer rates capture the link between nutrient management and crop growth^75^. The significance of these human-driven factors, as demonstrated in 2001, DCM proved to be significant in yield estimation, underscoring the importance of human factors.

Limitations of optical remote sensing, such as spectral mixing and confusion, can be mitigated by integrating multi-temporal and multi-dimensional data, including fluorescence for photosynthesis, temperature for growth stages, and soil moisture for water stress^76^. Combining these with agricultural modernization metrics like irrigation and planting density ensures a holistic representation of crop growth processes, enhancing prediction accuracy and robustness^77–79^.

### Deficiencies and prospects

Future research should develop efficient methods for high-precision crop yield estimation using minimal and accessible data sources. While remote sensing techniques often utilize phenological data for large-area yield estimation, acquiring such data remains challenging in regions with data scarcity or loss^80^. This study highlights the potential of incorporating agricultural modernization metrics like DCM and DFC, which are reliable and consistent, making them valuable for large-scale, long-term yield predictions. Expanding datasets to include phenological data and broader anthropogenic metrics is essential for improving model accuracy over larger regions and longer timescales^81^.

Despite the strong performance of CNNAtBiGRU in managing spatial heterogeneity and surpassing traditional models, biases persist. The model overestimates yields in southwestern grassland-sandy areas and western wetland-lake regions, while underestimating yields in northern forested areas under extreme climatic conditions. These biases likely arise from limited sample sizes in high-yield regions and reliance on average trend yields, which obscure county-specific variations^82,83^. Addressing these biases will require integrating higher-resolution remote sensing data, such as Landsat or Sentinel- 2, to better capture fine-scale crop condition variations, particularly in low-yield areas. Hybrid modeling approaches, such as combining CNNAtBiGRU with geographically weighted regression or attention-based frameworks, could enhance adaptability across agroecological zones. Ancillary datasets, including irrigation status, pest prevalence, and socio-economic factors, could further reduce residual biases^84^.

The transferability of CNNAtBiGRU to other regions or crops remains uncertain due to variations in climatic, soil, and management conditions. Adapting the model for broader applications may require modifying parameters, input variables, and training data for specific regions or crops with differing phenologies, soils, and practices. Variability in the availability and reliability of supporting datasets, such as mechanization and fertilizer usage rates, could also affect performance^85^. Future studies should test adaptability through cross-regional validation and extend applications to crops like wheat, rice, and soybean^86^.Domain-specific transfer learning could fine-tune the model using limited local data, while hybrid frameworks integrating CNNAtBiGRU with agro-climatic zoning or ensemble approaches could improve generalizability^87^. Collaboration with regional agricultural stakeholders for high-quality data and validation will be key to scaling the model for practical applications^88^.

In this study, MODIS data with a 500-meter resolution served as the primary input for yield estimation, balancing data availability, computational efficiency, and scalability. However, this resolution limits the model’s ability to capture fine-scale heterogeneity, especially in fragmented agricultural landscapes. The decision not to use higher-resolution datasets like Landsat or Sentinel was influenced by computational challenges associated with large-scale high-resolution data. Nevertheless, high-resolution imagery can enhance yield estimates by reducing spatial misclassification^89–91^. Future efforts should integrate higher-resolution datasets, leveraging advancements in cloud computing platforms such as Google Earth Engine and AI-based data fusion techniques to improve spatial granularity and performance, particularly in heterogeneous cropping systems^82^.

## Conclusion

This study introduced a novel crop yield estimation model combining bidirectional propagation, an attention mechanism, and agricultural modernization data. The model predicted early-season yields 1–2 months before the end of the growing season and yielded results with R² values exceeding 0.9 and RMSE values below 0.9 ton/ha for earlier years. However, its effectiveness depends on accurate maize classification masks. The increasing availability of high-resolution global satellite imagery from platforms like Landsat, Sentinel, and Planet, along with cloud-based processing advancements, enables detailed mapping of maize fields during the growing season. Incorporating real-time, high-resolution maize mapping data into the framework could improve spatial accuracy and timeliness, enhancing yield forecasting and supporting precision agriculture practices and decision-making on regional and global scales.

## Data Availability

The datasets used and analysed during the current study available from the corresponding author on reasonable request.
